# Transcriptomic events associated with internal browning of apple during postharvest storage

**DOI:** 10.1186/s12870-014-0328-x

**Published:** 2014-11-28

**Authors:** Ifigeneia Mellidou, Kim Buts, Darwish Hatoum, Quang Tri Ho, Jason W Johnston, Christopher B Watkins, Robert J Schaffer, Nigel E Gapper, Jim J Giovannoni, David R Rudell, Maarten LATM Hertog, Bart M Nicolai

**Affiliations:** Division of Mechatronics, Biostatistics and Sensors, Department of Biosystems (BIOSYST), KU Leuven, Willem de Croylaan 42, bus 2428, Leuven, 3001 Belgium; Flanders Centre of Postharvest Technology, Willem de Croylaan 42, Leuven, 3001 Belgium; Fruit Tree Research Laboratory, US Department of Agriculture/Agriculture Research Service, Wenatchee, WA 9880 USA; Department of Horticulture, Cornell University, Ithaca, NY 14853 USA; Boyce Thompson Institute for Plant Research, Cornell University, Ithaca, NY 14853 USA; The New Zealand Institute for Plant & Food Research Limited, Mount Albert Research Centre, Private Bag 92169, Auckland 1142 New Zealand; The University of Auckland, Private Bag 92019, Auckland, 1142 New Zealand; Plant, Soil, and Nutrition Laboratory, US Department of Agriculture/Agriculture Research Service, Ithaca, NY 14853 USA

**Keywords:** Apple fruit, Browning disorder, Metabolic pathways, Postharvest physiology, RNA sequencing, Transcriptomics

## Abstract

**Background:**

Postharvest ripening of apple (*Malus* x *domestica*) can be slowed down by low temperatures, and a combination of low O_2_ and high CO_2_ levels. While this maintains the quality of most fruit, occasionally storage disorders such as flesh browning can occur. This study aimed to explore changes in the apple transcriptome associated with a flesh browning disorder related to controlled atmosphere storage using RNA-sequencing techniques. Samples from a browning-susceptible cultivar (‘Braeburn’) were stored for four months under controlled atmosphere. Based on a visual browning index, the inner and outer cortex of the stored apples was classified as healthy or affected tissue.

**Results:**

Over 600 million short single-end reads were mapped onto the *Malus* consensus coding sequence set, and differences in the expression profiles between healthy and affected tissues were assessed to identify candidate genes associated with internal browning in a tissue-specific manner. Genes involved in lipid metabolism, secondary metabolism, and cell wall modifications were highly modified in the affected inner cortex, while energy-related and stress-related genes were mostly altered in the outer cortex. The expression levels of several of them were confirmed using qRT-PCR. Additionally, a set of novel browning-specific differentially expressed genes, including pyruvate dehydrogenase and 1-aminocyclopropane-1-carboxylate oxidase, was validated in apples stored for various periods at different controlled atmosphere conditions, giving rise to potential biomarkers associated with high risk of browning development.

**Conclusions:**

The gene expression data presented in this study will help elucidate the molecular mechanism of browning development in apples at controlled atmosphere storage. A conceptual model, including energy-related (linked to the tricarboxylic acid cycle and the electron transport chain) and lipid-related genes (related to membrane alterations, and fatty acid oxidation), for browning development in apple is proposed, which may be relevant for future studies towards improving the postharvest life of apple.

**Electronic supplementary material:**

The online version of this article (doi:10.1186/s12870-014-0328-x) contains supplementary material, which is available to authorized users.

## Background

After harvest, apples (*Malus* × *domestica* Borkh.) are typically stored under a controlled atmosphere (CA) with reduced O_2_ and increased CO_2_ levels to extend their commercial storage life. A major problem of several apple cultivars during CA storage is the development of internal browning disorders. Depending on the disorder, incidence can be aggravated by low storage temperatures and CA conditions, either high CO_2_, low O_2_, or a combination of the two. The ‘Braeburn’ apple cultivar is particularly susceptible to flesh browning, and at least two different expressions of the disorder have been identified. One is a dark discoloration that is initiated in the cortical flesh [1], while the other usually develops in the area near the seed cavities and may extend from the inner region near the core to the outer cortex (Additional file 1: Figure S1) [2].

Internal browning disorders have been extensively studied in pears [3-5], and apples [6,7]. Besides the various symptoms which may vary between species, cultivars, or CA conditions, browning can be associated with membrane damage resulting from stresses caused by low temperature, low O_2_ and/or elevated CO_2_ concentration during CA storage [7]. In gas-related disorders, oxidative stress can also be aggravated by the fruit geometry that induces additional gradients within the fruit, resulting in increased hypoxia towards the centre of the fruit [8-11]. This oxidative stress may in turn cause a shift of the cellular metabolism from the respiratory to the far less efficient fermentation pathway. As a result, less energy may become available to maintain membrane integrity under the constant stress of reactive oxygen species (ROS). Eventually, the loss of membrane integrity can lead to the disruption of cellular compartmentalisation, as has been shown by the leak of cellular liquid into the intracellular spaces, hence impeding diffusion of gases through tissue [7]. The release of phenolic compounds from the vacuole, and polyphenol oxidases (PPO) from the plastids, results in the enzymatic oxidation of phenols by PPOs to o-quinones and the formation of the brown-coloured pigment melanin [12]. A protective role for _L_-ascorbic acid (AsA) has also been proposed, due to its ability to reduce quinones back to precursor phenols [13,14].

The apple consensus genome (‘Golden Delicious’) has a relatively small size of ∼ 750 Mb and 63,541 predicted genes [15]. Several RNA-sequencing (RNA-Seq) studies have been recently reported on apple, investigating tree architecture [16,17], pathogen infection [18], and CO_2_ injury during postharvest storage [19]. The goal of this study was to explore the apple transcriptome changes associated with flesh browning during storage of ‘Braeburn’ apple. Based on recent studies [10,11], gas gradients at the currently applied CA storage conditions indicate that the main trigger for browning development is likely the very low oxygen concentration in the inner cortex (below 0.5%), and not the high CO_2_ concentrations as observed in other apple browning disorders.

In this manuscript, the RNA-Seq technology was used to explore transcriptomic events after four months of CA storage. Based on a visual browning index (BI), the inner and outer cortex of the stored apples was classified as healthy or affected tissue. Reads obtained were mapped against the *Malus* consensus coding sequence (CDS) set and browning-related differentially expressed genes (DEGs) were identified using multivariate statistical tools. Based on associations between expression levels of the candidate genes and browning incidence in fruit stored at various other storage conditions, potential biomarkers were suggested for assessing the risk of browning development.

## Results and discussion

To obtain an overview of the browning-related transcriptomic changes, cDNA libraries of inner and outer cortex samples from four individual fruits collected at harvest and from 16 individual fruits collected after four months storage were designed for Illumina RNA-Seq. Over 640 million short single-end reads were generated (Additional file 2: Table S1), with each cDNA library containing on average 16 million high-quality reads (after trimming for low quality bases and sequences of less than 20 nucleotides). The mean reads mapping rate was 70.8 ± 4.2% (Additional file 2: Table S1), of which 66.1 ± 0.6% mapped uniquely against the predicted gene set [15]. Only 29.2% of the reads were not counted in the RNA-Seq mapping process. This reads mapping rate is considerably higher than those reported by other authors working with *Malus* (35.8% of uniquely mapped reads according to [18]; 65% of total reads according to [19]). The total number of expressed genes was on average 30,816 ± 1,346 per sample or around 48.5% of the *Malus* predicted CDS set (Additional file 2: Table S1). A total of 25,287 and 22,464 expressed genes were found in all inner and outer cortex samples, respectively, with a total of 21,128 genes found in common (data not shown).

### Transcriptomic differences between healthy and affected tissues

The initial ‘Partial least squares discriminant analysis’ (PLS-DA) model containing all genes revealed poor discrimination between healthy (low BI) and affected (high BI) fruit cortex. However, the final reduced models after jack-knifing, were able to explain 96% and 84% of variance between the two classes (healthy-affected) in the inner (containing 578 DEGs) and outer (containing 1456 DEGs) cortex, respectively. These sets of DEGs were filtered for fold change between healthy and affected tissues of either >1.5 or < −1.5, resulting in 357 (inner) and 560 (outer) DEGs. Finally, the DEGs were filtered to exclude those genes significantly up- or down-regulated with time independent of the incidence of browning (Additional file 2: Table S2). These were identified by comparing fruit at harvest to the healthy fruit after storage under the assumption these DEGs were more generally related to ripening. This resulted in the final set of 234 and 459 browning-specific DEGs in the inner and in the outer fruit cortex, respectively (Additional file 2: Tables S3, S4). Only five genes were in common when comparing DEGs from the inner and the outer cortex. Specifically, a disease resistance protein (MDP0000153857), a cyclin kinase (MDP0000722904), and an eukaryotic translation initiation factor (MDP0000378642) were induced in both cortex tissues of affected apples, whereas a nac domain-containing protein (MDP0000207408) and an uncharacterized protein (MDP0000299891) were repressed in the affected tissues. This limited overlap in transcriptomic events is indicative of the spatial differences in the regulation of browning potentially related to the spatial variation in gas conditions inside the fruit [7,11].

**Figure 1:**
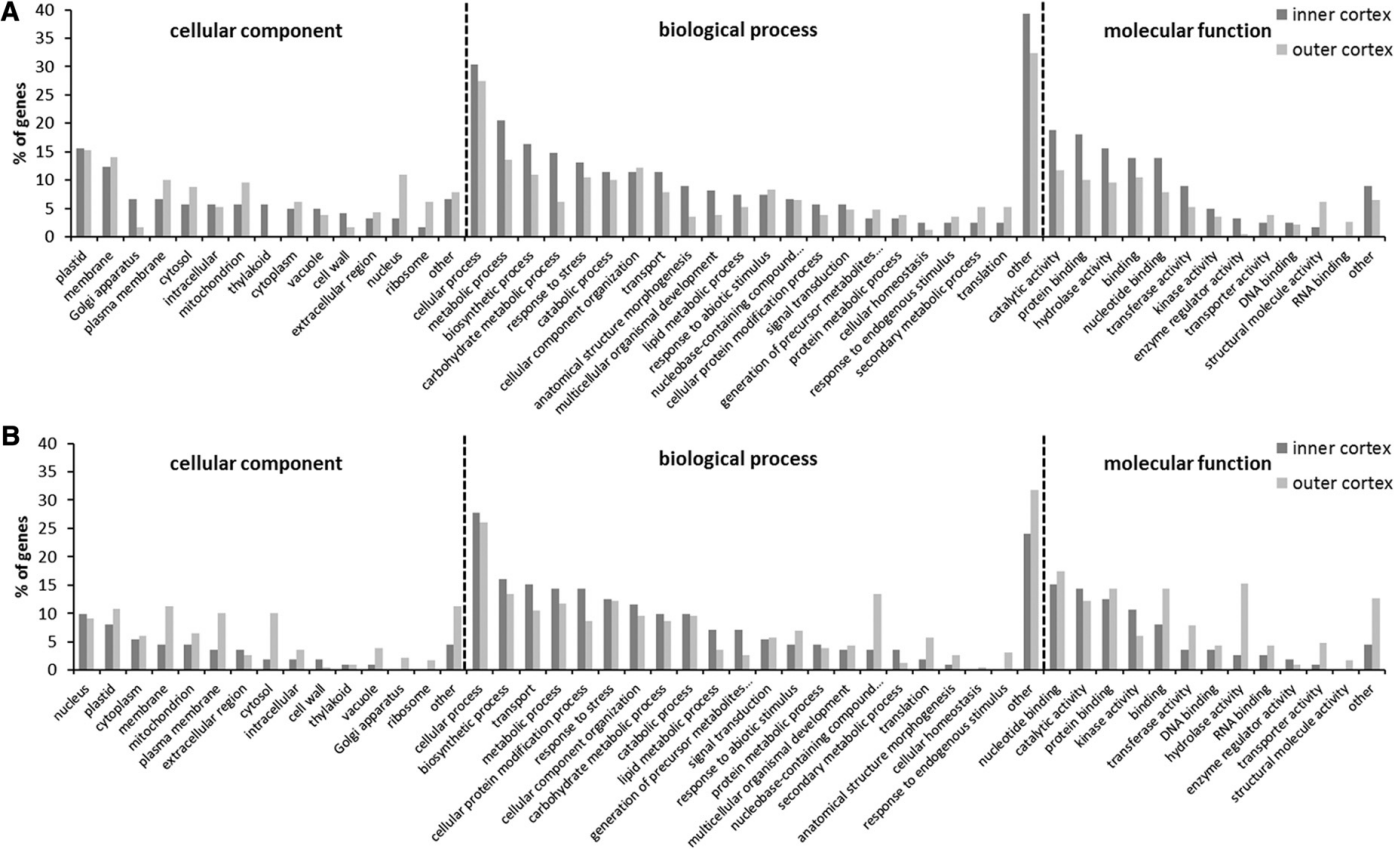
**GO functional classifications of the apple transcripts.** Cellular component, biological process, and molecular function classifications of DEGs in the inner (dark grey) and outer (light grey) cortex that are induced **(A)** or repressed **(B)** in affected tissues.

GeneOntology (GO) analyses returned a blast hit for over 85.5% of the genes, and GO terms could be assigned to over 70% of these genes (data not shown). Over-representations of GO terms in the set of DEGs in affected tissues were evaluated to indicate which biological processes, molecular functions and cellular components were mostly affected by the disorder (Figure 1). Several significantly induced GO terms representing cellular components were associated with plastids and membranes for both affected inner and outer cortex (Figure 1A). This comes to no surprise as plastids in fruits are involved in fatty acid (FA) and isoprenoid synthesis, and in the generation of non-photosynthetic ATP and reducing power [20]. The biological processes significantly enriched in the set of induced DEGs in the affected inner cortex were the cellular (30.3%) and metabolic processes (20.5%). Other over-represented categories of biological processes included the biosynthetic (16.4%), and carbohydrate metabolic processes (14.8%), and stress responses (13.1%). A significant set of DEGs were also related to the lipid metabolic process (7.38%), signal transduction (5.74%) and the generation of precursor metabolites and energy (3.28%). Similar results were obtained for the outer cortex, with the main difference being the lower percentage of DEGs related to carbohydrate metabolic processes (6.11%). The GO terms for molecular function up-regulated in affected inner and outer cortex included genes coding for proteins with catalytic or hydrolase activity, and for binding proteins (i.e., nucleotide-, protein-, zinc-, ATP-binding). The set of DEGs with hydrolase activity included several genes involved in the hydrolysis of membrane-related lipids/phospholipids (Tables 1 and 2).

**Table 1:** **Browning-related genes induced or repressed in the affected inner cortex of ‘Braeburn’ apples**

				**RPKM**		
***Malus*** **ID**	**Gene description**	**Function**	**p-value**	**Healthy**	**Affected**	**Fold change**
**Energy-related pathways**						
MDP0000192364	Pyruvate dehydrogenase	Link glycolysis to TCA	<0.001	14.53	29.39	2.02
MDP0000129305	Phosphoglycerate mutase	Glycolysis/dually targeted	0.016	0.91	1.80	1.98
MDP0000295823	Probable lactate/malate dehydrogenase	Fermentation	<0.001	20.06	30.24	1.51
MDP0000622920	Phosphoenolpyruvate carboxylase	TCA/org transformation	0.004	8.35	5.47	−1.53
MDP0000198410	Aconitate hydratase	TCA/org transformation	0.016	19.75	11.72	−1.69
MDP0000798440	Cytochrome c biogenesis fn	Mitochondrial electron transport/ATP synthesis	0.014	2.67	1.52	−1.76
**Lipid metabolism**						
MDP0000249250	Phospholipase a2	Lipid degradation	0.004	0.49	4.25	8.65
MDP0000576682*	Butyrate-- ligase peroxisomal-like	FA synthesis and FA elongation	0.01	0.20	1.30	6.63
MDP0000794484	Alpha/beta-Hydrolase	Lipid degradation	0.003	2.60	6.33	2.43
MDP0000833444	Diacylglycerol kinase-like	Phospholipid synthesis	0.018	2.14	5.15	2.41
MDP0000309977	Acyl-coenzyme a thioesterase	Lipid degradation/beta-oxidation	0.004	0.95	2.23	2.36
MDP0000849585	Alpha/beta-Hydrolase	Lipid degradation	0.009	2.85	5.69	2.00
MDP0000283158	Alpha beta-hydrolase, lipase	Lipid degradation	<0.001	4.51	7.92	1.76
MDP0000273425	Diacylglycerol kinase-like	Phospholipid synthesis	0.04	4.44	2.73	−1.63
**Redox state**						
MDP0000615196	Glutaredoxin family protein	Redox state	<0.001	0.32	1.18	3.64
MDP0000146621	Nadh-cytochrome b5 reductase	Redox state	0.004	4.39	8.03	1.83
MDP0000196554	Chorismate mutase/APX	Aminoacid synthesis/ascorbate metabolism	<0.001	11.63	19.96	1.72
MDP0000251669	Thioredoxin	Redox state	0.019	18.00	28.15	1.56
MDP0000252195	Ferredoxin-thioredoxin reductase	Redox state	<0.001	10.84	16.70	1.54
MDP0000520089*	Desacetoxyvindoline 4-	Ascorbate and glutathione metabolism	0.041	5.41	3.53	−1.53
MDP0000181414	Alpha-ketoglutarate-dependent dioxygenase	Redox state	0.004	6.69	4.16	−1.61
**Secondary metabolism**						
MDP0000260512	4-coumarate: ligase	Phenylpropanoids	0.001	0.16	1.05	6.71
MDP0000576682*	Butyrate-- ligase peroxisomal-like	Sulfur-containing glucosinolates synthesis	0.01	0.20	1.30	6.63
MDP0000206680	Reticuline oxidase-like	Alkaloid-like	<0.001	1.48	5.13	3.45
MDP0000702557*	UDP-glucuronosyl/UDP-glucosyltransferase	Flavonoids/flavonols synthesis	0.001	30.37	48.24	1.59
MDP0000520089*	Desacetoxyvindoline 4-	Sulfur-containing glucosinolates synthesis	0.041	5.41	3.53	−1.53
**Cell wall**						
MDP0000610961	L-ascorbate oxidase	Cell wall modifications	<0.001	30.35	132.43	4.36
MDP0000873667	Xyloglucan endotransglucosylase/hydrolase	Cell wall modification	<0.001	52.59	229.40	4.36
MDP0000904458	Fasciclin-like arabinogalactan	Cell wall proteins	<0.001	193.78	525.70	2.71
MDP0000723275	Arabinose 5-phosphate isomerase	Cell wall precursor synthesis	<0.001	18.80	32.91	1.75
MDP0000289339	Cellulose synthase	Cell wall cellulose synthesis	0.013	1.90	1.03	−1.85
MDP0000836165	Pectin methylesterase inhibitor	Cell wall pectin esterases	0.036	2.42	1.21	−2.00
MDP0000616949	Pectin methylesterase inhibitor	Cell wall pectin esterases	0.01	1.04	0.44	−2.37

P-values for DEGs between healthy and affected apples were calculated using PLS-DA, where gene expression values (RPKM) were used as predictor variables and the two class distinctions as response variables. Blast2GO and Mercator web tools were used for gene description and gene function analysis.

*Genes that have been assigned to more than one metabolic pathways.

**Table 2:** **Browning-related genes induced or repressed in the affected outer cortex of ‘Braeburn’ apples**

				**RPKM**		
***Malus*** **ID**	**Gene description**	**Function**	**p-value**	**Healthy**	**Affected**	**Fold change**
**Energy-related pathways**						
MDP0000158797	Bisphosphoglycerate-independent phosphoglycerate mutase	Glycolysis cytosolic branch	0.015	1.67	4.25	2.54
MDP0000149088	Ubiquinone biosynthesis protein coq4	Mitochondrial electron transport/ATP synthesis	0.01	3.59	6.55	1.82
MDP0000251581	Succinate dehydrogenase	TCA/org transformation	0.003	25.49	42.53	1.67
MDP0000376244	Pyruvate kinase	glycolysis cytosolic branch	0.006	80.59	125.23	1.55
MDP0000807498	Cytochrome b-c1 complex	mitochondrial electron transport/ATP synthesis	0.008	32.39	49.30	1.52
MDP0000134766	Ubiquinol-cytochrome c reductase	mitochondrial electron transport/ATP synthesis	0.016	33.28	50.20	1.51
MDP0000163886	Aconitate hydratase	TCA/org transformation	<0.001	34.74	23.14	−1.50
MDP0000141199	Malate dehydrogenase	TCA/org transformation	0.01	68.62	45.82	−1.50
MDP0000581903	Glyceraldehyde 3-phosphate dehydrogenase	Glycolysis cytosolic branch	0.012	142.56	93.35	−1.53
MDP0000120718	atp-citrate synthase	TCA/org transformation	<0.001	82.25	53.31	−1.54
MDP0000631825	Pyruvate kinase isozyme chloroplastic-like	Glycolysis plastid branch	0.005	13.34	8.44	−1.58
MDP0000168246	atp-citrate synthase	TCA/org transformation	0.016	11.77	7.05	−1.67
MDP0000743397	Pyruvate kinase cytosolic	Glycolysis cytosolic branch	0.039	12.05	6.33	−1.90
MDP0000119941	Dihydrolipoyl dehydrogenase	*PDH* complex	0.048	3.46	1.76	−1.97
MDP0000677354	Alcohol dehydrogenase	Fermentation	<0.000	7.30	3.32	−2.20
MDP0000186461	Alcohol dehydrogenase	Fermentation	<0.001	5.52	2.33	−2.37
MDP0000746317	Coenzyme Q biosynthesis coq4	Mitochondrial electron transport/ATP synthesis	0.003	2.04	0.72	−2.85
**Lipid metabolism**						
MDP0000235803	Phospholipase c	Lipid degradation	0.026	0.17	1.13	6.56
MDP0000293806	Acyl CoA oxidase	Lipid degradation/beta-oxidation	0.049	0.75	1.34	1.78
MDP0000270312	Neutral/alkaline non-lysosomal ceramidase	Exotics' (steroids, squalene etc.) sphingolipids	0.002	2.81	4.71	1.68
MDP0000847523	Acyl-CoA thioesterase	Lipid degradation/beta-oxidation	0.008	5.30	8.86	1.67
MDP0000190112	Serine C-palmitoyltransferase	Exotics' (steroids, squalene etc.) sphingolipids	0.047	1.36	2.27	1.66
MDP0000084546*	Cycloartenol synthase	Exotics' (steroids, squalene etc.)	0.002	1.47	2.21	1.51
MDP0000209755	Enoyl CoA hydratase	Lipid degradation/beta-oxidation	0.007	4.52	2.71	−1.67
MDP0000422184	Sphingosine-1-phosphate lyase	Exotics' (steroids, squalene etc.) sphingolipids	0.028	10.76	6.40	−1.68
**Redox state**						
MDP0000508761	Flavonol synthase flavanone 3-hydroxylase-like	Redox state/flavonoid biosynthesis	0.015	0.57	1.27	2.23
MDP0000364366	Superoxide dismutase	Redox state/dismutases and catalases	0.016	19.44	30.52	1.57
MDP0000699607	Catalase	Redox state/dismutases and catalases	0.035	53.52	80.67	1.51
MDP0000217438	L-galactose-1-phosphate phosphatase	Ascorbate biosynthesis	0.006	17.14	11.24	−1.52
MDP0000203927	Glutathione peroxidase	Ascorbate and glutathione metabolism	<0.001	49.07	22.51	−2.18
**Secondary metabolism**						
MDP0000639264	3 -n-debenzoyl-2 -deoxytaxol n-benzoyltransferase	Phenylpropanoids	0.005	1.48	2.64	1.79
MDP0000312032	3-hydroxy-3-methylglutaryl coenzyme a reductase	isoprenoids/mevalonate pathway	0.009	21.49	32.33	1.50
MDP0000269612	Cinnamoyl- reductase	phenylpropanoids/lignin biosynthesis	0.002	210.28	315.08	1.50
MDP0000157996	3-hydroxy-3-methylglutaryl coenzyme a reductase	isoprenoids/mevalonate pathway	0.018	3.72	1.94	−1.92
**Cell wall**						
MDP0000785413	Alpha-expansin	Cell wall modification	0.004	2.25	5.26	2.34
MDP0000259640	Alpha-expansin	Cell wall modification	0.008	2.67	4.92	1.85
MDP0000171438	Auxin-repressed protein, pectin lyase	Cell wall pectin esterases	0.04	8.22	13.47	1.64
MDP0000162976	Pectinacetylesterase	Cell wall pectin esterases	0.014	57.35	86.41	1.51
MDP0000314627	cellulose synthase	cell wall cellulose synthesis	0.004	13.03	8.05	−1.62

P-values for the differentially expressed genes between healthy and affected apples were calculated using PLS-DA, where gene expression values (RPKM) were used as predictor variables and the two class distinctions as response variables. Blast2GO and Mercator web tools were used for gene description and gene function analysis.

*Genes that have been assigned to more than one metabolic pathways.

Under the cellular component category, many repressed DEGs in affected tissues were categorized as nucleus (9.8% in inner, 9.1% in outer cortex) or plastids (8.0% in inner, 10.9% in outer cortex), but in contrast to the set of induced DEGs, several differences were observed between the inner and the outer cortex (Figure 1B). In particular, genes associated to membrane, cytosol or vacuole seemed to be more repressed in the affected outer than the affected inner cortex. The top most abundant biological process categories significantly down-regulated in affected tissues included the cellular process (27.7% in inner, 26.1% in outer cortex), the biosynthetic process (16.1% in inner, 13.5% in outer cortex) and transport (15.2% in inner, 10.4% in outer cortex). The most notable down-regulation of molecular functions in affected tissues were the numerous GO terms related to kinase activity.

**Figure 2:**
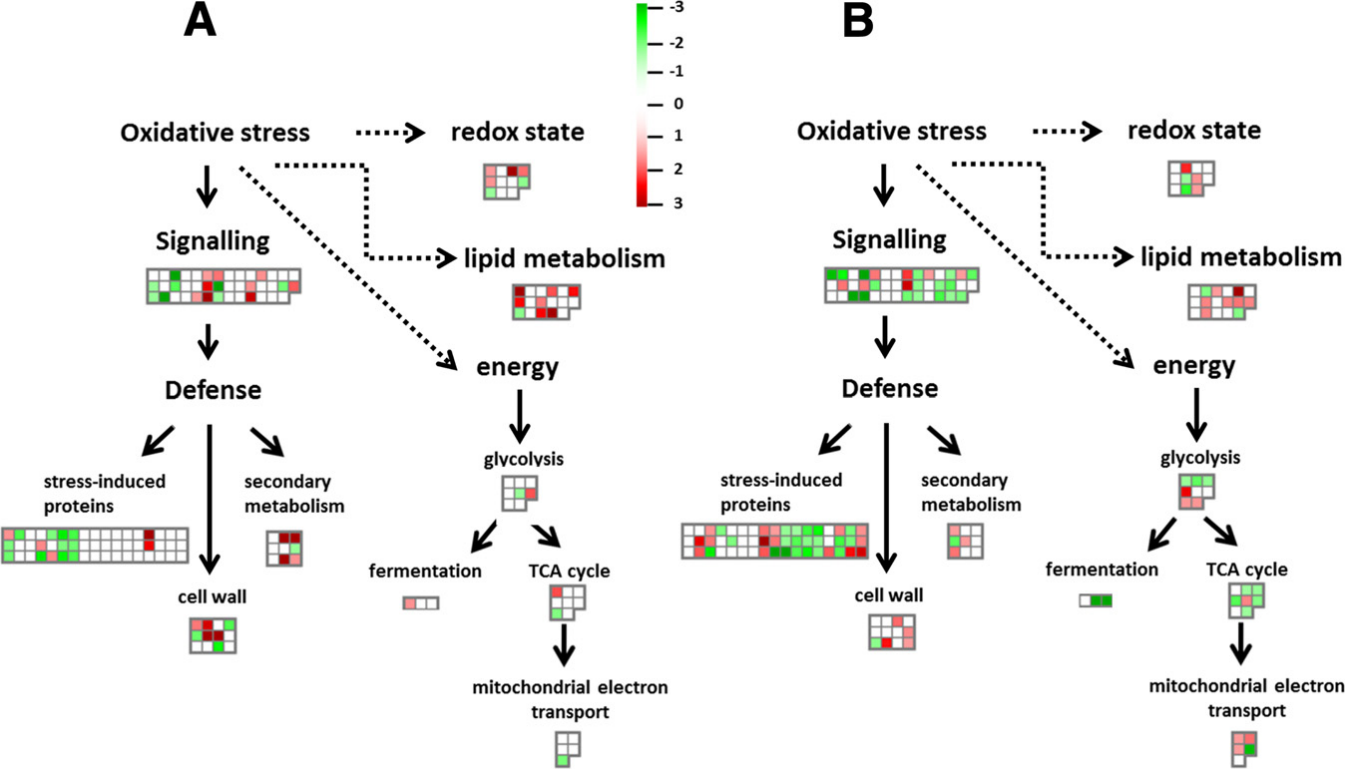
**MapMan overview of DEGs from selected pathways between healthy and affected tissues** **(A.** **inner cortex;** **B.** **outer cortex).** Induced genes in affected tissues are indicated in red and repressed genes in green. The scale bar displays changes in gene expression as fold change that were significant (p < 0.05) between the two class distinctions as indicated by PLS-DA. ABA: abscisic acid, TCA: tricarboxylic acid.

**Figure 3:**
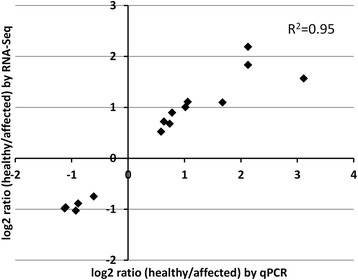
**Correlation between RNA-Seq and qRT-PCR (as log2 ratio of relative expression of healthy/affected tissue).** The relative expression levels of the selected genes (Additional file 2: Table S5) were obtained by RNA-Seq data and by qRT-PCR. The Pearson correlation coefficient is shown.

The overall distribution of DEGs in the 35 MapMan bins/pathways is summarized in Additional file 1: Figure S2, whereas an overview of the metabolic changes occurring in affected apple cortex is shown in Additional file 1: Figure S3. The most abundant DEGs were involved in stress (6.67% in inner, 7.23% in outer cortex), signalling (6.67% in inner, 4.82% in outer cortex), transport (4.31% in inner, 4.62% in outer cortex), cell (4.71% in inner, 4.02% in outer cortex), lipids (3.14% in inner, 1.81% in outer cortex), energy (1.95% in inner, 3.60% in outer cortex), and redox state (2.35% in inner, 1.00% in outer cortex) pathways. The discussion of these results focuses on pathways expected to be involved or influenced by browning development at CA conditions, such as energy-related, lipid metabolism, cell wall modifications, redox state, and secondary metabolism (Figure 2, Tables 1 and 2). Furthermore, a close correlation (R^2^ = 0.95) was observed between log2-fold changes measured by RNA-Seq and real-time quantitative PCR (qRT-PCR; Figure 3) on a selection of 15 DEGs (Additional file 2: Table S5), indicating that fold-change values obtained from sequencing are accurate.

### Candidate genes for browning development

**Figure 4:**
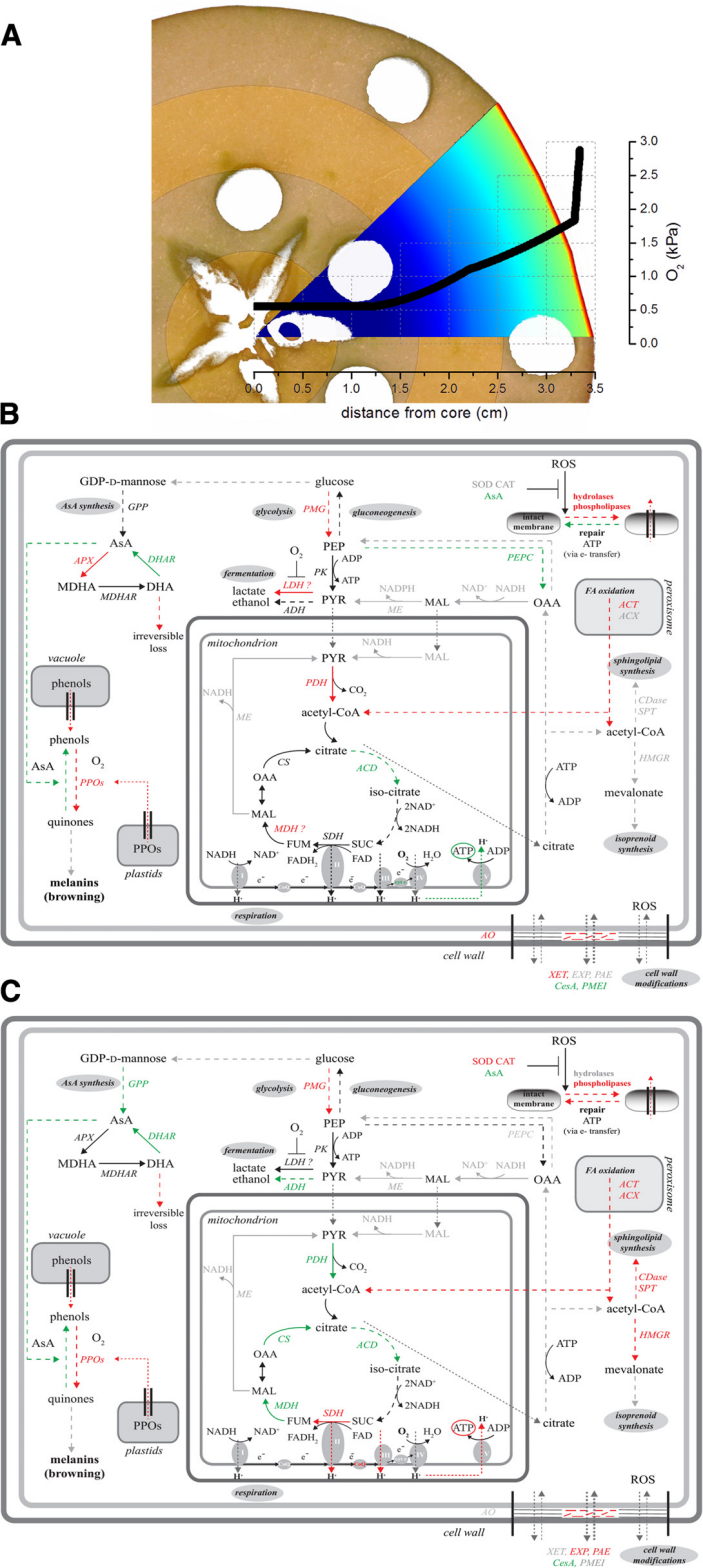
**Proposed model for browning development in apples during CA storage. A**. Distribution of O_2_ for a typical ‘Braeburn’ apple exposed to CA storage conditions (3 kPa O_2_ and 0.7 kPa CO_2_) as simulated by [11]. **B-C**. Transcriptomic changes in the affected inner **(B)** and outer **(C)** cortex related to the gas gradient and energy potential. Induced genes in affected tissues are indicated in red, repressed genes in green, and unchanged genes in grey. AsA: ascorbic acid; *ACD*: aconitase dehydratase; *ACT*: acyl CoA thiolesterase; *ACX*: acyl CoA oxidase; *ADH*: alcohol dehydrogenase; *AO*: ascorbate oxidase; *APX*: ascorbate peroxidase; *CesA*: cellulose synthase; *CAT*: catalase; *CDase*: ceramidase; CoQ: coenzyme Q10; *CS*: citrate synthase; DHA: dehydroascorbate; *DHAR*: dehydroascorbate reductase; *EXP*: expansin; FUM: fumarate; *GPP*: m-galactose-1-phosphate phosphatase; *HMGR*: 3-hydroxy-3-methylglutaryl-CoA reductase; *LDH*: lactate dehydrogenase; MAL: malate; *MDH*: malate dehydrogenase; MDHA: monodehydroascorbate; *ME*: malic enzyme; *MDHAR*: monodehydroascorbate reductase; OAA: oxaloacetate; *PAE*: pectinacetylesterase; *PDH*: pyruvate dehydrogenase; PEP: phosphoenolpyruvate; *PEPC*: phosphoenolpyruvate carboxylase; *PMG*: phosphoglyceratemutase; *PK*: pyruvate kinase; *PMEI*: pectin methylesterase inhibitors; *PPO*: polyphenol oxidase; PYR: pyruvate; *SDH*: succinate dehydrogenase; *SOD*: superoxide dismutase; *SPT*: serine-palmitoyltransferase; SUC: succinate; *XET*: xyloglucanendotransglucosylase/hydrolase. *LDH* and *MDH* are followed by a question mark as MDP0000295823 encodes a putative lactate/malate dehydrogenase protein.

The different transcriptomic responses in the inner and outer cortex should be interpreted against the spatially different gas conditions inside the fruit. Due to the fruit’s geometry and the properties of the different fruit tissues (e.g., peel, and cortex), internal gas gradients will develop [7,9,10]. As a result hypoxic stress will increase when moving from the outer towards the inner cortex. Based on the work presented by [11], simulations were performed for a typical ‘Braeburn’ apple exposed to the currently applied CA storage conditions (3% O_2_ and 0.7% CO_2_) indicating that the expected O_2_ levels at the position of the outer and inner cortex were around 1.6% and 0.5% respectively (Figure 4A). Additionally, the expected CO_2_ levels at the inner and outer cortex were around 0.7% and 1.1%, respectively (data not shown), suggesting that CO_2_ was not the trigger for the development of the disorder. Given these expected gas gradients, the identified DEGs are discussed separately to highlight tissue-specific responses to low O_2_ stress.

#### Genes involved in energy-related pathways

Plant cells synthesize energy-rich molecules like ATP and reductive power (NADH) via pathways such as photosynthesis, glycolysis, the tricarboxylic acid (TCA) cycle, and the mitochondrial electron transport chain (ETC.). An inhibition or a deficiency in one or more intermediates of these pathways may cause a wide range of metabolic disturbances leading to postharvest disorders. In this study, several DEGs between healthy and affected tissues were linked to the energy-related pathways. This may be related to the loss of membrane integrity due to the prolonged exposure to low O_2_ levels at low temperature (discussed below). Under such circumstances, cells need to synthesize protective compounds to maintain their cellular compartmentalisation, and to detoxify metabolic intermediates accumulated. Indeed, the RNA-Seq results provided evidence of changes in all parts of the respiratory pathways of affected tissues (Tables 1 and 2; Figure 2), with the different cortex locations balancing their energy needs in a different manner.

Apart from the synthesis of ATP and NADH, glycolysis also serves to supply pyruvate to the mitochondrial TCA cycle. Overall, RNA-Seq data reveals a putative induction of glycolysis only in the affected inner cortex. Given the inner cortex of the fruit is exposed to lower O_2_ levels as compared to the outer cortex (Figure 4A), this is in agreement with recent findings from Ampofo *et al.* (personal communication) showing a clear up-regulation of the glycolysis in tomato cells when lowering O_2_ levels. This was interpreted as a cellular effort to maintain the overall energy supply in spite of the hypoxic stress limiting the ATP yield of the ETC. Phosphoglyceratemutase (*PMG*), previously proposed as the key gene controlling glycolysis in potato [21], showed significant induction in both the affected inner (MDP0000129305) and outer cortex (MDP0000158797). No other glycolytic genes were changed in the affected inner cortex, whereas the expression of an abundant pyruvate kinase (*PK*, MDP0000376244) was increased in the affected outer cortex. Nevertheless, two other members of the same gene family (cytosolic, MDP0000743397; plastid, MDP0000631825) were significantly repressed, indicating the complex regulation of the pathway in different organelles. The highly abundant glyceraldehyde 3-phosphate dehydrogenase (MDP0000581903) was only repressed in the affected outer cortex. Putative fermentation-related genes were induced (1.5-fold) in the affected inner cortex (lactate/malate dehydrogenase, MDP0000295823), but repressed more than 2.2-fold in the affected outer cortex (alcohol dehydrogenase, *ADH*; MDP0000186461, MDP0000677354). This is in agreement with the expected O_2_ profiles in apple with the stronger hypoxia in the inner cortex triggering anaerobic metabolism.

Under aerobic conditions, the respiratory mechanism continues with the TCA cycle reactions in two ways. First, pyruvate produced in glycolysis can be transported to mitochondria where it is irreversibly oxidized to acetyl-CoA and CO_2_ by pyruvate dehydrogenase (PDH). This gene is thought to be the key step to regulate fluxes through the TCA cycle [22]. Secondly, phosphoenolpyruvate is converted to malate and/or pyruvate by cytosolic phosphoenolpyruvate carboxylase (PEPC), and then transferred to mitochondria. In the affected inner cortex, *PDH* (MDP0000192364) was significantly induced and *PEPC* (MDP0000622920) repressed, suggesting an induction of the first path towards the TCA cycle. Although none of these genes changed significantly in the outer cortex, a putative dihydrolipoyl dehydrogenase (MDP0000119941), which is part of the PDH complex, was repressed. Acetyl-CoA enters the TCA cycle by condensation with oxaloacetate to form citric acid, which is ‘recycled’ back to oxaloacetate in a series of successive reactions, with the concomitant production of flavin adenine dinucleotide (FADH_2_) and NADH. With lowering O_2_ levels, TCA slows down, and glycolysis becomes the sole source of energy until the activation of fermentation. Indeed, the results suggested a down-regulation of the TCA cycle, particularly at the outer cortex (Figure 2). Specifically, aconitase hydratase (MDP0000198410, MDP0000163886) was significantly repressed at both cortex locations. As this enzyme is assumed to be sensitive to oxidative stress and regulated by iron availability [23], it could serve as a 'stress marker'. On the other hand, malate dehydrogenase (*MDH*, MDP0000141199), and citrate synthase (*CS*, MDP0000168246, MDP0000120718), were only repressed in the outer cortex. Both *MDH* and *CS* can be inhibited by oxidized lipids such as polyunsaturated FAs generated under oxidative stress [24].

The reducing power produced through the previous steps can be used in the ETC. to drive ATP synthesis. Succinate dehydrogenase (*SDH*) plays a dual role in both the TCA cycle and the ETC. Knock-out mutations in plants resulted in far-reaching perturbations in organic acids levels, photosynthesis, respiration rates and mitochondrial ROS generation [25]. An abundant *SDH* (MDP0000251581) was significantly induced in the affected outer cortex (Table 2), but remained unchanged in the inner part. Kinetic results indicated that *SDH* depends on the ubiquinone reduction levels, and is activated by ATP [26]. An ubiquinol-cytochrome c reductase (MDP0000134766) was indeed induced in the affected outer cortex, whereas another two genes from the mitochondrial ETC. (ubiquinone biosynthesis protein coenzyme Q10, MDP0000149088; cytochrome b-c1 complex, MDP0000807498) were also up-regulated (Table 2). By contrast, the ETC. in the affected inner cortex was possibly down-regulated (repression of a cytochrome c biogenesis protein, MDP0000798440), suggesting that less energy was available to maintain membrane integrity.

#### Genes involved in lipid metabolism

During long-term CA storage, fruits need sophisticated mechanisms to tolerate oxidative stress, to guarantee ample energy production and to maintain membrane integrity. In total, 16 lipid-related DEGs, most of them encoding key enzymes of lipid degradation pathways, were identified in the affected tissues (Tables 1 and 2). Changes were more severe in the inner cortex as indicated by the higher fold-change (Figure 2). This confirms the membrane lipid alterations in the affected tissue similar to pears [27].

Phospholipids serve as signal transduction molecules under stress conditions, such as cold and hypoxia [28]. Phospholipase a2 (inner, *PLA2*, MDP0000249250) and phospholipase c (outer, MDP0000235803) were among the top up-regulated genes, with affected tissues having 8.7- or 6.6-fold higher expression than healthy tissues, respectively. Alterations in the expression of phospholipases may have activated phospholipid signalling in response to CA-induced stress. Additionally, alpha/beta-hydrolases (MDP0000794484, MDP0000849585 and MDP0000283158) were induced in the affected inner cortex, probably indicating the more extended alterations in membrane integrity occurring there.

Peroxisomal FA beta-oxidation has multiple roles in plants, generating the substrate (acetyl-CoA) for the synthesis of isoprenoids, flavonoids, and FAs, as well as providing the respiratory substrate under carbohydrate-depleted stress conditions [29]. An acyl-CoA thioesterase (*ACT*) was induced in both tissues (inner, MDP0000309977; outer, MDP0000847523), whereas an acyl-CoA oxidase (*ACX*, MDP0000293806) was only induced in the outer cortex. These results indicate a possible induction of the peroxisomal FA oxidation in the affected tissues at CA storage affecting lipid turnover.

Two other lipid-related genes, involved in FA synthesis (butyrate-ligase, MDP0000576682) and in phospholipid synthesis (diacylglycerol kinase-like, MDP0000833444) were significantly up-regulated in the affected inner cortex. By contrast, two genes involved in the sphingolipid metabolism (neutral/alkaline non-lysosomalceramidase, MDP0000270312; serine C-palmitoyltransferase, MDP0000190112) and a gene of the steroid biosynthetic pathway (cycloartenol synthase; MDP0000084546) were significantly induced in the affected outer cortex. Sphingolipids comprise a major class of lipid signalling molecules in all eukaryotic cells having roles in mediating programmed cell death associated with plant defence [30]. Only few genes were down-regulated in the affected tissues, including a diacylglycerol kinase (MDP0000273425) in the inner cortex, as well as an enoyl CoA hydratase (MDP0000209755) and a sphingosine-1-phosphate lyase (MDP0000422184) in the outer cortex.

#### Genes involved in redox state

Internal browning in apple involves multiple oxidation-reduction processes and accumulation of various antioxidant enzymes to cope with the overproduction of ROS induced by the low O_2_ stress. Seven DEGs were assigned as ROS scavengers in the inner cortex, including large families such as thioredoxins (MDP0000251669), glutaredoxins (MDP0000615196), and ferredoxin-thioredoxin reductases (MDP000025219), and five in the outer cortex, including glutathione peroxidase (MDP0000203927).

One of the most intriguing DEG in the inner cortex was a chorismate mutase (*CM*; MDP0000196554), which, apart from functioning in the aromatic amino acid synthesis, also has ascorbate peroxidase (APX) activity. Ascorbate peroxidase participates in the ascorbate-glutathione cycle scavenging H_2_O_2_ and recycling AsA in the cells [31]. Here, the expression of *APX* was high in the affected inner cortex, suggesting that the defence path has been triggered by the ROS burst. Nevertheless, if the AsA recycling pathway does not work efficiently, once oxidized to dehydroascorbic acid (DHA), AsA is no longer available. The current results showed a significant decrease in seven (inner cortex) or five (outer cortex) out of the ten *Malus* dehydroascorbate reductase (*DHAR*) genes with the rest remaining unchanged (Additional file 2: Table S6), indicating the malfunction of the cycle, and thus the incapacity to properly recycle AsA. *DHAR* has been identified as the key gene linked to susceptibility to flesh browning after cut [32]. Consequently, AsA content is expected to be low in both cortex locations. Low fruit AsA content has been associated to an increased susceptibility to browning in apples [33] and pears [3]. The importance of the AsA metabolism is further supported by the enhanced _L_-galactose-1-phosphate phosphatase (*GPP*, MDP0000217438) in the healthy outer cortex. This gene is involved in the main AsA biosynthetic pathway via _L_-galactose, and is considered as a key step of the pathway under stress in tomato fruit [34].

Superoxide dismutase (SOD) is an important antioxidant enzyme catalysing the dismutation of superoxide to O_2_ and H_2_O_2_. Meanwhile, H_2_O_2_ can be either indirectly scavenged by the AsA recycling pathway, or it can be directly reduced to water and O_2_ by catalase (CAT). Here, the expression of the abundant *SOD* (MDP0000364366) and *CAT* (MDP0000699607) was significantly up-regulated in the affected outer cortex to withstand the high ROS burst, dissimilar to the inner part where browning has already progressed and cell death processes may have been irreversibly triggered. Recent proteomic studies on apple ripening also demonstrated that SOD may have a major role in the redox state system during ripening and senescence [35]. When considering the whole fruit, the up-regulated DEGs in the affected outer cortex may be good indicators of browning incidence in the inner part of the fruit.

#### Genes involved in secondary metabolism

Secondary metabolism (SM) plays a key role in the protection of plants against (a)biotic stresses. Several genes related to SM showed differential expression during storage (e.g. chorismate mutase, peroxidases; Additional file 2: Tables S3-S4), suggesting a shift from the primary to the SM and possible perturbations in the overall fruit metabolism and signal-transduction. Genes related to the major SM classes (phenylpropanoids, terpenoids/isoprenoids, and alkaloids/glucosinolates) were differentially expressed in affected tissues (Tables 1 and 2), but the induction was higher in the inner cortex (Figure 2). Through the phenylpropanoid pathway, several defence-related metabolites can be produced, including flavonoids, and lignins. A 4-coumarate: ligase (MDP0000260512), which catalyses the last step of the phenylpropanoid pathway leading either to lignins or to flavonoids, was significantly induced (6.7-fold) in the inner cortex. By contrast, an abundant cinnamoyl-reductase (MDP0000269612) from the lignin biosynthetic pathway was induced in affected outer cortex. Although the link between an induction of lignification and internal browning is poorly understood, a negative correlation between lignin content and browning incidence of apple fruit infected by *Penicillium expansum* has been reported [36].

The first committed and rate-limiting step in the mevalonate pathway for isoprenoid biosynthesis is catalysed by 3-hydroxy-3-methylglutaryl-CoA reductase (HMGR), and modulated by many endogenous and external stimuli [37]. The abundant *HMGR* (MDP0000312032) was significantly induced in the outer affected cortex, whereas another one with low expression [reads per kilobase of exon per million mapped reads (RPKM) <4] was repressed (MDP0000157996). It has been reported that the induction of *HMGR* during storage can be involved in the accumulation of α-farnesene in apple skin, a compound related to the post-harvest disorder of superficial scald [38]. Other gene expressions altered in the affected tissues were related to flavonoid, glucosinolates or alkaloid synthesis (Tables 1 and 2), although their exact function is still poorly understood. The overexpression of genes related to flavonoid accumulation can be explained as the effort of affected tissues to balance out the oxidative stress synthetizing compounds with antioxidative properties, as already reported during the development of apple superficial scald [39].

#### Genes involved in cell wall modifications

A clear interaction between fruit softening and browning development was indicated in this study, such that a higher up-regulation of the cell wall modification paths occurred in the affected inner cortex (Figure 2). Ascorbate oxidase (AO) is an apoplastic enzyme linked to cell wall modifications, controlling the redox state of the apoplastic AsA pool and regulating stress perception and signal transduction [40]. An abundant *AO* (MDP0000610961) was significantly induced (4.4-fold) in the affected inner cortex, suggesting a role in browning. The product of apoplastic oxidation of AsA by AO, DHA, is transported to the cytosol, where it can be recycled by the ascorbate-glutathione cycle [31]. Given that *DHAR* was down-regulated (Additional file 2: Table S6), it is suggested that AsA may be irreversibly oxidized, enabling browning to occur.

Several genes involved in cell wall loosening were induced in affected tissues. Xyloglucanendotransglucosylase/hydrolase (*XET*; MDP0000873667), that is involved in the breakdown of hemicelluloses [41], was induced in the affected inner cortex. The activity of this enzyme coincides with the initial slow softening phase of postharvest apple fruit softening [42]. Alpha-expansin (*EXP*; MDP0000785413, MDP0000259640) also showed high expression in the affected outer cortex. An increased expression of both *XET*s and *EXP*s has been implicated in aril breakdown of longan (*Dimocarpus longan*) fruit stored at low temperature [43]. Two cell wall pectin esterases, an auxin-repressed pectin-lyase (MDP0000171438) and a pectinacetylesterase (MDP0000162976) were induced in the affected outer cortex. Pectatelyases catalyse the eliminative cleavage of de‐esterified pectin, and have multiple functions, not only in pathogen attack, but also in various developmental processes including ripening and softening [44]. Apart from cell wall loosening related genes, a fasciclin-like arabinogalactan (MDP0000904458) and an arabinose 5-phosphate isomerase (MDP0000723275) were also induced in the affected inner cortex. Fasciclin-like arabinogalactans are genes involved in cell adhesion and cell expansion, and are highly expressed in immature fruit [45], while the function of arabinose 5-phosphate isomerase in eukaryotes is still poorly understood.

Genes involved in cell wall synthesis, and in particular cellulose synthesis (cellulose synthase, *CeAs*), were repressed in both affected inner (MDP0000289339) and outer (MDP0000314627) cortex. It was demonstrated that a decrease in the expression of *CesA* in the early developmental stages of fruit growth, together with an increase in the expression of *EXP*s, are key regulators of cell wall biosynthesis during apple fruit development [46]. Additionally, two pectin methylesterase inhibitors (*PMEI*, MDP0000836165, and MDP0000616949) were down-regulated in the affected inner cortex, suggesting their putative role during abiotic stress exposure (e.g., cold, anoxia). *PMEI*s are cell wall proteins that regulate the activity of pectin methymesterases, and control the spatial distribution of esterified pectins in fruit [47].

#### Genes involved in ethylene biosynthesis

In agreement with previous studies in pear [4], a 1-aminocyclopropane-1-carboxylate oxidase (*ACO*; MDP0000200896) was down-regulated in the affected inner cortex as compared to the healthy tissue. ACO catalyses the last step in the ethylene biosynthetic pathway, in a reaction that needs both O_2_ and AsA as substrate. A reduced *ACO* expression in ‘Golden Delicious’ apples during CA storage has been reported [48], while high levels of H_2_O_2_ can inactivate ACO activity [49]. As such, the low expression of the specific *ACO* gene copy observed in this study only in the affected inner cortex, can be explained by: 1) the severe hypoxic conditions in the inner part of the fruit compared to the outer part, 2) the possible increased amounts of H_2_O_2_, and/or 3) the reduced AsA content. The expression of *ACO* is expected to be repressed by the treatment with 1-methylcyclopropene (1-MCP), as applied in this study. Nevertheless, the fact that the specific *ACO* was only repressed in the affected inner cortex, and that its expression did not change over time, suggest that the expression of this gene copy is related to the disorder, and not to 1-MCP as such. Furthermore, the putative role of *ACO* as a ‘browning-biomarker’ has been confirmed in stored apples not treated with 1-MCP (see validation below). The expression of the other highly abundant *ACO* members, MDP0000195885 (chr10) and MDP0000200737 (chr5), did not differ between healthy and affected outer cortex (data not shown), suggesting a putative role of the *MdACO* presented here in the development of browning disorder in ‘Braeburn’ that should be further investigated.

#### The role of polyphenol oxidases

Regardless the cause of flesh browning (enzymatic after cutting or physiological due to stress), the eventual brown discolouration is usually the result of interactions between PPO activity and polyphenol contents. As discussed, the alterations in membrane permeability occurring during CA storage facilitates the release of both enzyme and substrate in the cytosol leading to the formation of brown pigments. As such, internal browning development under CA conditions could be explained by the compartmentalisation and activity of PPOs. A correlation between PPO activity and the incidence of internal browning has been previously reported in pineapple [50]. Additionally, reduced PPO activity may contribute to resistance against scald development in apples [51]. In this study, two *PPO*s were induced in both the inner (*MdPPO-1*, MDP0000249183) and the outer (MDP0000539552) cortex (Additional file 2: Tables S3-S4). In fact, *MdPPO-1* was one of the most abundant transcripts found with RPKM values >1700 (Additional file 2: Table S3). Flesh browning in minimally processed apples has been linked to an induction of *PPO* expression [52]. Although browning occurred earlier than the induction of *PPO* expression, the authors postulated that *PPO* was activated to stimulate the polyphenol signalling system playing an important role in the plant’s defence against pests and insects [53]. In the end, the tissue breakdown induced by the abiotic stress of CA storage is not that different of similar defects induced by insect herbivory or microbial invasions thus triggering the same universal plant defence response.

### Putative biomarkers for browning disorder

**Figure 5:**
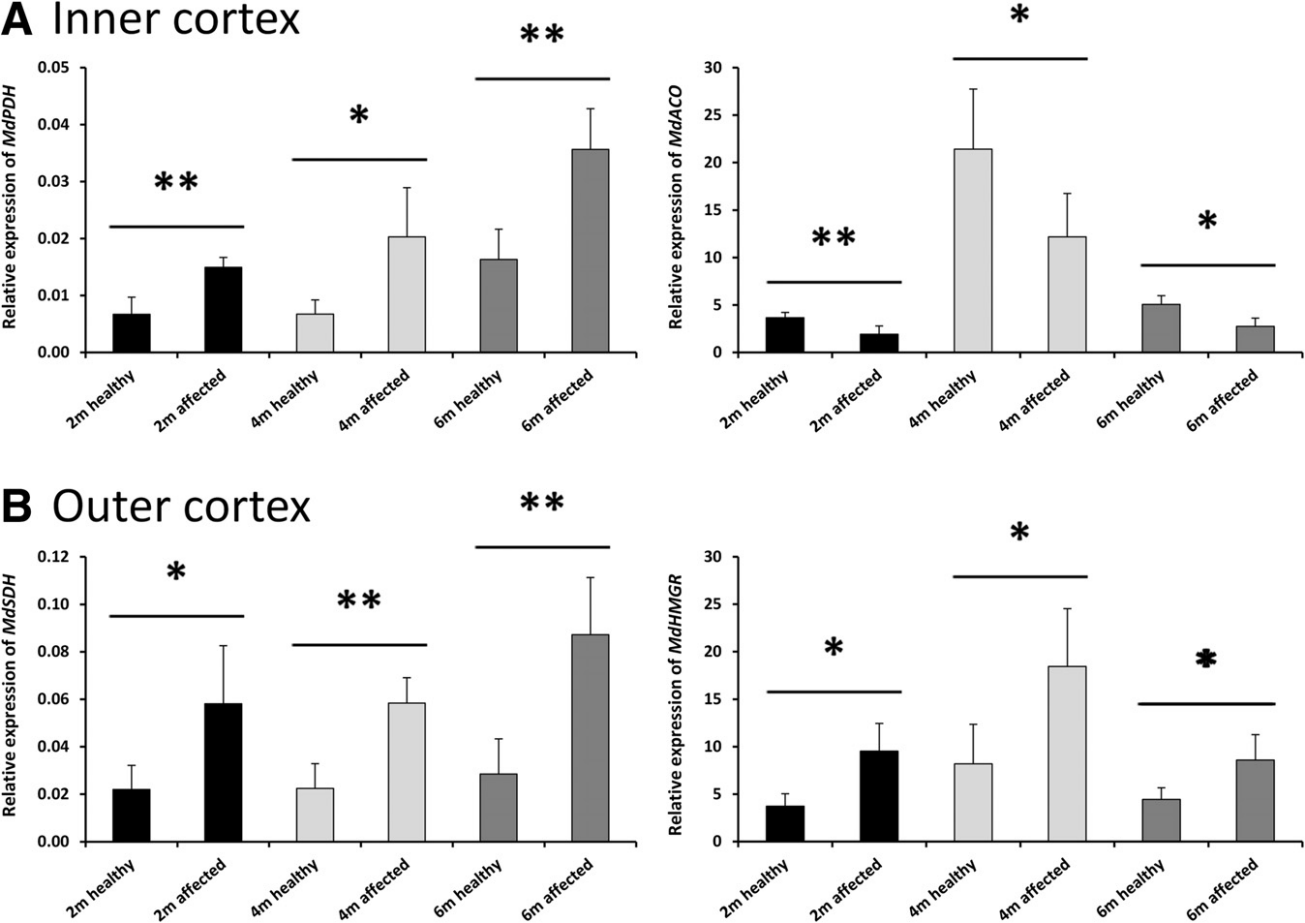
**Gene expression studies using qRT-PCR.** Relative expression of pyruvate dehydrogenase (*MdPDH*; MDP0000192364), and 1-aminocyclopropane-1-carboxylate oxidase (*MdACO*; MDP0000200896) in the inner cortex **(A)**, and succinate dehydrogenase (*MdSDH*; MDP0000251581) and 3-hydroxy-3-methylglutaryl coenzyme a reductase (*MdHMGR*; MDP0000312032) in the outer cortex **(B)** of ‘Braeburn’ apples stored for two, four, or six months under various CA conditions. qRT-PCR values were normalized against the geometrical mean of ubiquitin (MDP0000154072 ) and actin (MDP0000886327). The error bars represent standard deviation of five biological replications. Significance of the mean relative gene expression of each group was tested with Student’s t-test (*P < 0.05, **P < 0.01) using the SAS software.

Using multivariate statistics to compare healthy and affected tissues with various degrees of browning development (from initiation to more severe stages), putative biomarkers to predict the incidence of browning were identified (Tables 1 and 2). Of them, 15 genes (Additional file 2: Table S5) were randomly selected for verification by qRT-PCR 1) on the same sample set as the RNA-Seq, and 2) on another ‘validation’ set that contained fruits stored for various periods at different CA conditions to test their broader potential (Figure 5, Additional file 1: Figure S4). Of the selected genes, *MdPDH* (glycolysis) and *MdACO* (ethylene metabolism) were the only genes that were significantly induced or repressed, respectively, across all selected time points, in the affected inner cortex (Figure 5). Similarly, *MdSDH* (mitochondrial ETC.) and *MdHMGR* (mevalonate pathway) were induced under the various conditions in the affected outer cortex. The other selected genes were only validated in one or two experiments (Additional file 1: Figure S4).

Interestingly, all the DEGs identified through the RNA-Seq analysis in the inner cortex were validated at four month storage, except for *MdCesA*. The difference between the RNA-Seq experiment and the qRT-PCR validation set was the application of 1-MCP and the lowest CO_2_ concentration in the first experiment. Most of the DEGs were also validated at CA storage for six months (except for *MdPLA2*), whereas only a few were verified at CA storage for two months (browning-inducing conditions at high CO_2_ levels). Nevertheless, there was a clear trend in agreement to the RNA-Seq results, suggesting that despite the different trigger (gas conditions), similar metabolic pathways are altered in the affected inner cortex. By contrast, DEGs between healthy and affected outer cortex were not verified in the validation set. Exceptions included *MdPK* and *MdPPO-2* that were validated as significant DEGs at six or four months, respectively. These findings, in combination with the observation that significant DEGs in the inner cortex were stable in the outer cortex (and *vice-versa*), as well as the limited overlap between the different cortex locations, clearly suggest a spatial variation in the regulation of browning induction, most likely related to the spatial O_2_ distribution.

### What makes apples turn brown?

Prolonged postharvest storage at CA conditions can induce cellular oxidative stress by promoting ROS production and accumulation, thus causing oxidation of cellular components and loss of organelle integrity. The oxidative stress is expected to be more severe in the inner cortex, where the O_2_ concentration is around 0.5%, compared to the 1.5-2% in the outer cortex (Figure 4A). Based on the presented RNA-Seq results, a conceptual model for browning development is proposed (Figure 4B-C).

The changes in the redox state within affected tissues were evident in the whole fruit. In an attempt to control the excessive generation of ROS, several antioxidant-related genes were induced, including *APX*, glutaredoxins and thioredoxins (inner cortex), or *CAT* and *SOD* (outer cortex). Up-regulation of the ROS scavenging mechanism could have served as the trigger for the changes in metabolic and energy-related pathways. To cope with abiotic stress induced by the prolonged exposure to low temperature and low O_2_, a continuous energy supply to control ROS production and maintain membrane integrity, is vital. A clear induction of glycolytic pathway occurred in the affected inner cortex (*PMG*), suggesting that pyruvate supply is not the rate-limiting factor, while the overall picture in the affected outer cortex was less clear with multiple DEGs being both induced and repressed. An increased flux to the TCA cycle occurred in the inner cortex (up-regulation of *PDH*), perhaps as an attempt to restore the energy potential. The TCA cycle itself was repressed in both tissues, and as such the rate of oxidative phosphorylation should be induced to maintain the energy balance. Indeed, the up-regulation of the ETC. in the outer cortex may have counteracted the effects of the down-regulated TCA cycle. By contrast, oxidative phosphorylation in the inner cortex was disturbed presumably as a result of the ultra-low O_2_ conditions (0.5%), and, therefore, an alternative energy production path via fermentation may have been activated (induction of a putative lactate/malate dehydrogenase). Fermentation-related genes such as *ADH* were repressed in the affected outer cortex. On the other hand, peroxisomal degradation of FAs, mediated by the β-oxidation cycle, help produce energy and acetyl-CoA. Up-regulation of FA oxidation occurred in both tissues, although an induction of acetyl-CoA related pathways (including mevalonate pathway and sphingolipid synthesis) only occurred in the outer cortex. Consequently, it is proposed that the energy status in the outer cortex may still efficiently balance the harmful effects of ROS accumulation presumably via increased energy production, whereas the reduced energy generation in the inner cortex may be insufficient to adverse the damage.

Less energy means incapacity to repair membranes, which, particularly in the inner cortex, are damaged as a result of induction of a/b hydrolases and phospholipases. In turn, loosening of cellular compartmentalisation allows the release of *PPO*s and phenols to the cytosol, triggering the actual discolouration reactions. Affected tissues showed higher *PPO* expression serving as a generic plant defence response [52]. The effect of increased PPO activity can be reversed by AsA [14]. However, considering 1) the induction of AsA oxidation (inner cortex; high *APX* and apoplastic *AO*) and 2) the repression of AsA biosynthesis (outer cortex; low *GPP*) in the affected tissues, as well as 3) the repression of AsA recycling capacity (both; low *DHAR*) due to storage, it is postulated that AsA content is lower in the affected fruit. The changes in the redox state of the AsA pool may have served as the signal for other stress responses to hypoxic stress.

During browning development in stored apples, genes involved in cell wall loosening including *AO*, *XET*, *PLAs* and *EXPs* are mostly induced, while genes involved in cell wall synthesis or strengthening, such as *CeA*s or *PMEI*s, are mostly repressed. This observation suggest for the first time that susceptibility to postharvest disorders such as internal browning are somehow linked to cell wall modifications, similar to those observed during fruit softening, or the response to biotic stress. This may be also linked to the formation of cavities observed at severely affected apples. Apart from the lack of overlap between the sets of DEGs identified in each cortex location as a response to the oxidative stress, several pathways were strongly altered in a tissue-specific manner (Figure 2). Specifically, genes involved in lipid metabolism, SM, and cell wall modifications were highly modified in the affected inner cortex, while stress-related or energy-related genes were mostly altered in the outer cortex. This is presumably due to the spatial distribution of O_2_ induced by the fruit’s microstructure [7], and the resulting gas exchange properties (Figure 4A).

## Conclusions

This is the first report of a complete quantitative transcriptome analysis of internal browning disorder in apples stored under CA conditions revealing numerous novel regulatory candidate genes, and confirming results from proteomic approaches in other pome fruit, such as pear. Results indicated alterations in several metabolic pathways occurring during postharvest storage of apple, including the repression of the TCA cycle, as well as the up-regulation of the ETC. and the FA oxidation. These alterations can be seen as an attempt to control the excessive generation of ROS induced by the storage conditions eventually lead to browning development in a tissue-specific manner. The RNA-Seq results provided here may serve as a ‘genetic’ roadmap of fruit-tissue specific changes to postharvest-induced oxidative stress, clearly suggesting spatial differences in the regulation of browning. This study can serve as starting point for additional proteomic and metabolomic screenings to create a complete view on the development of the disorder at the various ‘omic’ levels. Further validation is required to assess the importance of the proposed candidate genes as potential markers to predict the risk of browning incidence in apples during CA storage.

## Methods

### Plant material and postharvest conditions

Trees of cv ‘Braeburn’ (clone Hillwell), grafted on a M9 rootstock, were grown in the orchard of the experimental tree fruit research station (RSF-PCfruit) in Sint-Truiden, Belgium. Apples were harvested on October 26, 2011, the commercial picking date for long-term storage of ‘Braeburn’ as determined by the Flanders Centre of Postharvest Technology. Apples were stored for four months at 1°C under CA of 3% O_2_ and 0. 7% CO_2_ receiving commercial application of 1-MCP. The 1-MCP (SmartFreshTM, AgroFresh Inc. Spring House, PA, USA) treatment was done by exposing the fruits to 1-MCP (625 ppb) in airtight containers for 24 h. Under the specified CA conditions, a sufficient variability in the occurrence of visible browning symptoms was detected.

### Fruit sampling and browning assessment

In total, 20 individual fruit were sampled for the sequencing experiment. Four fruit were sampled at harvest, and after four of months storage another 16 fruit were sampled. In each one of them, 1 cm thick slices were cut through the equator of the fruit. From each fruit, five tissue samples of 1 cm diameter were taken from the inner and the outer cortex (Additional file 1: Figure S1), immediately frozen, crushed in liquid nitrogen and stored at −80°C. To assess browning, pictures were taken from the complementary tissue slice immediately after cutting using a digital camera and controlled light conditions. For each tissue sample a visual BI was calculated using an in-house developed MATLAB program (Matlab R2010, The MathWorks, Inc., Natick, MA, USA) as described previously [2]. The colour scale was based on ten classes ranging from yellow (one) to brown (ten). Tissues were classified as healthy (low BI) or affected (high BI) (Additional file 1: Figure S1; Additional file 2: Table S7). Three groups of apples were identified: 1) those with both healthy inner and outer cortex, 2) those with only inner cortex affected, and 3) those with both inner and outer cortex affected. Tissues from all three groups of apples were taken to cover the initiation and development of the disorder in a tissue-specific manner.

### RNA extraction, library construction, and sequencing

Total RNA was extracted from all 20 fruit (4 replicate fruit collected at harvest and 16 after storage) from both inner and outer cortex tissue samples as shown in Additional file 1: Figure S1. The replicate fruit collected after storage were further classified as healthy or affected, as shown in Additional file 2: Table S1. Ground tissue samples (300 mg) were homogenized in 800 μL of extraction buffer containing cetyl-trimethyl-ammonium bromide, according to [54]. The mixture was incubated at 65°C for 10 min with occasional mixing by inversion. Chloroform (800 μL) was added and mixed by inversion, and the mixture was centrifuged at 14,000 rpm for 10 min at room temperature. The supernatant was mixed with a half volume of ethanol, loaded and washed through the RNeasy mini kit column (Qiagen). The purity of total RNA extracted was determined in NanoDrop 2000 (Thermo Scientific), and the integrity was checked by electrophoresis (Gel Doc EZ Imager; Bio-Rad). Strand specific mRNA-Seq libraries were made using 2 μg of DNase-treated total RNA and T4 RNA ligase 1 adenylated adapters [55]. The purified libraries were quantified and 20 ng of each used for sequencing. Samples were sequenced on an Illumina HiSeq2000 (Illumina, San Diego, CA) at the Weill Medicine School Sequencing Facility (Cornell University, NY, USA) with a 51-bp single-end read length. Libraries were spread over four lanes, avoiding biological replicates in the same lane. The sequence files were generated using the Illumina pipeline in software CASAVA v1.8 in Sanger FASTQ format and are available in the European Nucleotide Archive (http://www.ebi.ac.uk/ena/data/view/PRJEB6096).

### Data processing and differential gene expression analysis

Quality control of the short single-end raw reads for sequence contaminants was performed using the Fastqc module in Galaxy (http://main.g2.bx.psu.edu/) [56]. Reads were then analysed using the RNA-Seq analysis tools of the CLC Genomics Workbench software v6.5 (CLC bio, Aarhus, Denmark), and mapped against the *Malus* consensus CDS set (http://www.rosaceae.org/). No more than two mismatches per read were allowed. Only unique mappings were counted for expression analyses.

Gene expression levels were normalized using RPKM values. The RPKM values of the 63,541 genes of healthy and affected, inner and outer cortex, were explored with principal component analysis to detect outliers using The Unscrambler (v10.1, Camo, Trondheim, Norway). Two outlier samples were detected and removed. The PLS-DA in combination with an iterative jack-knifing procedure was performed on the inner and outer cortex samples separately, to identify significant DEGs between healthy and affected tissues. Specifically, RPKM values were used as predictor variables and the class distinction (healthy-affected) as response variables. Through cross validation, using the uncertainty test with the optimal number of factors, a set of the most significant predictor variables was selected. This iterative process was repeated until the percentage of variability explained by the reduced set of genes no longer improved. The resulting set of DEGs was filtered based on the p-value for beta-coefficients for the model (p < 0.05) and on the fold change (>1.5 or < −1.5) in RPKM between the two classes. To ensure that the final gene selection only contained browning-related transcripts and not the more general ripening or storage-related ones, DEGs also showing up in healthy apples when comparing harvest to four month storage were removed from the dataset (Additional file 2: Table S2).

For each DEG, the latest GO annotation vocabulary for cellular component, biological process and molecular function, was obtained using Blast2GO v.2.7.0 [57]. Default parameter settings were applied and the GOslim option was set to reduce the number of functional classes. In addition, DEGs were uploaded to the Mercator webtool (http://mapman.gabipd.org/web/guest/app/mercator) to assign bincode mapping [58], and visualized using MapMan (v.3.6, http://mapman.gabipd.org/web/guest/mapman) [59]. Default parameters were retained, and Blast cut-off was set at 50.

### Technical and functional validation

In total, 15 candidate genes differentially expressed between affected and healthy fruit were selected for qRT-PCR validation making sure to cover multiple metabolic pathways. Specific primers (Additional file 2: Table S5) were designed using Primer3 web tool (http://bioinfo.ut.ee/primer3/) and verified against the *Malus* predicted consensus gene set using the BLAST function of the Genome Database for *Rosaceae*. The transcriptional profiles of the selected genes were analysed by qRT-PCR using the SYBR Green I technology on a Rotor Gene Q (Qiagen). The purified RNA used for RNA-Seq was reverse transcribed into cDNA using the QuantiTect Reverse Transcription Kit (Qiagen). All qRT-PCR reactions contained 500 ng/μl of cDNA template, 7.5 mL of Absolute QRT-PCR SYBR Green Mix (Thermo Fisher Scientific), and 0.25 μM primer pairs, in a final volume of 15 μl. The same cycling conditions, melting curve analysis and criteria of acceptance for reaction efficiency were applied as described previously [60]. All expression data were normalized against the geometric mean of the expression of two stable reference genes, ubiquitin (MDP0000154072) and actin (MDP0000886327).

The selected candidate genes were independently validated on apples (cv ‘Braeburn’) stored for two, four and six months under various CA conditions (Additional file 2: Table S8). RNA isolation, cDNA synthesis, and qRT-PCR analysis were performed on samples from the inner and outer cortex of these independent fruit as described above. Based on their BI (Additional file 2: Table S8), tissues were classified as healthy and affected, and the DEGs between the two class distinctions (*p < 0.05, **p < 0.01 and ***p < 0.001) were identified based on the t-test using the Statistical Analysis Software (SAS Enterprise Guide 4.2; SAS Institute Inc.).

### Availability of supporting data

The data sets supporting the results of this article are available in the European Nucleotide Archive (http://www.ebi.ac.uk/ena/data/view/PRJEB6096).

## Additional files

Additional file 1
**Figure S1.** Cross-section of apples with various degress of browning incidence. **Figure S2.** MapMan bins. **Figure S3.** Overview of metabolic pathways of differentially expressed genes between healthy and affected tissues in MapMan. **Figure S4.** Validation of RNA-Seq results by qRT-PCR. Additional file 2
**Table S1.** Reads and their mapping on the *Malus* consensus sequences for inner and outer cortex. **Table S2.** Storage-related genes that were excluded from the analysis of RNA-Seq of browning disorder. **Table S3.** Differentially expressed genes between healthy and affected inner cortex. **Table S4.** Differentially expressed genes between healthy and affected outer cortex. **Table S5.** Primer sequences used for validation of RNA-Seq results by qRT-PCR. **Table S6.** Gene expression values (RPKM) of the different dehydroascorbate reductases (*DHAR*) as revealed by RNA-Seq.** Table S7.** The browning index (BI) of inner and outer cortex of apples used for RNA-Seq. **Table S8.** The browning index (BI) of inner and outer cortex of the validation set.

## Abbreviations

*ACO*: 1-aminocyclopropane-1-carboxylate oxidase
*ACT*: Acyl-CoA thioesterase
*ACX*: Acyl-CoA oxidase
*ADH*: Alcohol dehydrogenase
*AO*: Ascorbate oxidase
*APX*: Ascorbate peroxidase
AsA: _L_-ascorbic acid
BI: browning index
CA: Controlled atmosphere
*CAT*: Catalase
CDS: Coding sequences
*CM*: Chorismate mutase
*CeAs*: Cellulose synthase
*CS*: Citrate synthase
DEGs: Differentially expressed genes
DHA: Dehydroascorbate
*DHAR*: Dehydroascorbate reductase
ETC: Electron transport chain
*EXP*: Alpha expansin
FA: Fatty acids
GO: Gene ontology
*GPP*: L-galactose-1-phosphate phosphatase
*HMGR*: 3-hydroxy-3-methylglutaryl-CoA reductase
*MDH*: Malate dehydrogenase
*PDH*: Pyruvate dehydrogenase
*PEPC*: Phosphoenolpyruvate carboxylase
*PK*: Pyruvate kinase
*PLA2*: Phospholipase a2
PLS-DA: Partial least squares discriminant analysis
*PMEI*: Pectin methylesterase inhibitor
*PMG*: Phosphoglyceratemutase
*PPO*: Polyphenol oxidase
RNA-Seq: RNA sequencing
ROS: Reactive oxygen species
RPKM: Reads per kilobase of exon per million mapped reads
*SDH*: Succinate dehydrogenase
SM: Secondary metabolism
*SOD*: Superoxide dismutase
TCA: Tricarboxylic acid
*XET*: Xyloglucanendotransglucosylase/hydrolase
