# Supplementary material for: Transcriptomic events associated with internal browning of apple during postharvest storage
Source: BMC Plant Biol. 2014 Nov 28;14:328. doi: 10.1186/s12870-014-0328-x (PMC4272543; doi:10.1186/s12870-014-0328-x)
Supplement: Additional file 1 — Figure S1. Cross-section of apples with various degress of browning incidence. Figure S2. MapMan bins. Figure S3. Overview of metabolic pathways of differentially expressed genes between healthy and affected tissues in MapMan. Figure S4. Validation of RNA-Seq results by qRT-PCR. [file 12870_2014_328_MOESM1_ESM.pdf]

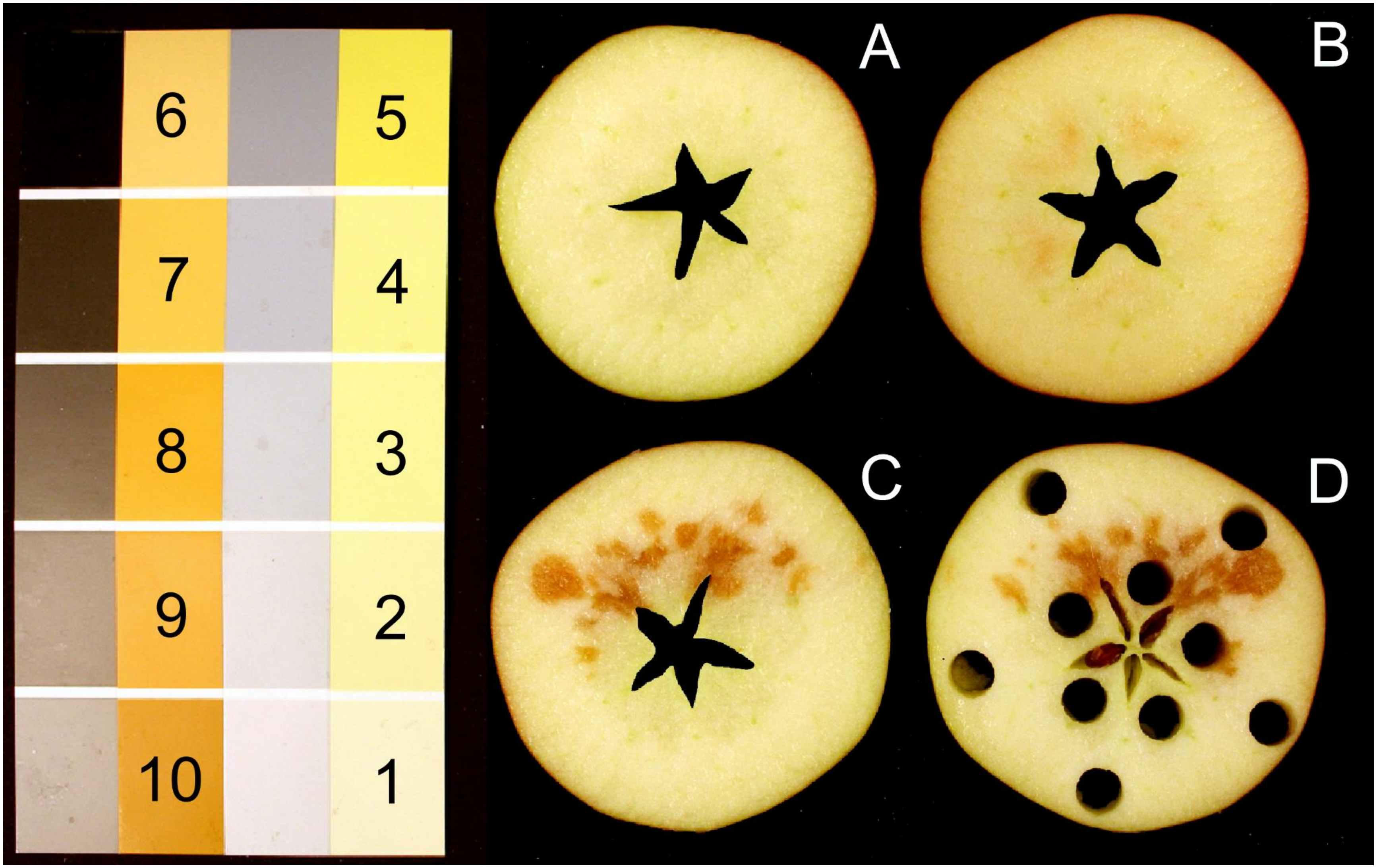

Figure S1. Cross-section of apples with various degrees of browning incidence. A: both inner and outer cortex are healthy. B: inner cortex is affected but outer cortex is still healthy. C: both inner and outer cortex are affected. D: cross section of an apple where the position of the inner cortex and outer cortex samples were taken from. The calibration colour card (1-10) was used to correct for the lightness of each image.

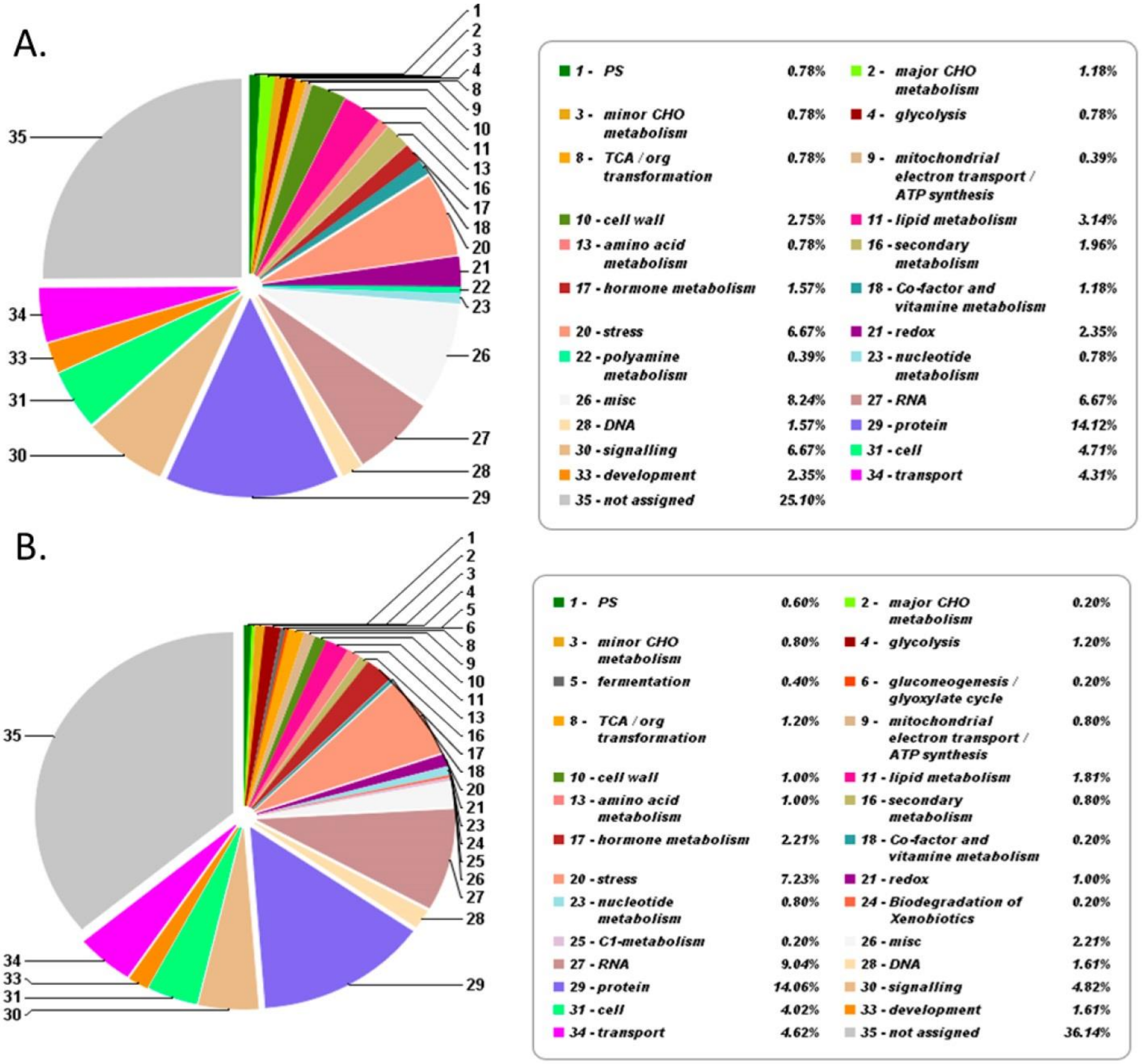

Figure S2. MapMan bins. Distribution of the differentially expressed genes of the inner (A) and outer (B) cortex between healthy and affected tissues stored for four months at CA conditions as obtained by Mercator webtool. DEGs were identified by PLS-DA analysis in The Unscrambler.

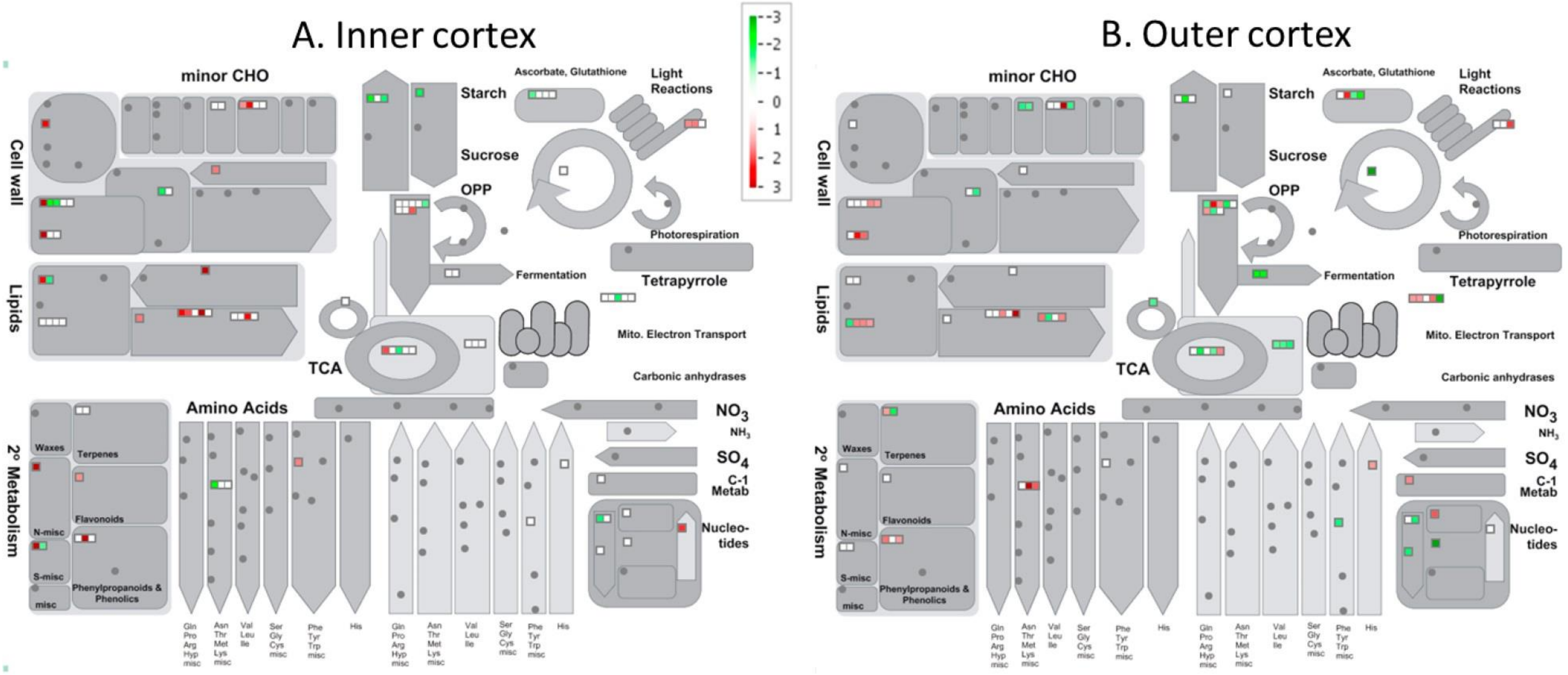

Figure S3. Overview of metabolic pathways of differentially expressed genes between healthy and affected tissues in MapMan. A. inner cortex; B. outer cortex. Apples were stored for four months at CA conditions. Genes that were induced in affected tissues are indicated in red and genes that are repressed in green. The scale bar displays changes in gene expression as fold change that were significant ($p<0.05$) between the two class distinctions as indicated by PLS-DA analysis. CHO: carbohydrates; OPP: oxidative pentose phosphate; TCA: tricarboxylic acid cycle.

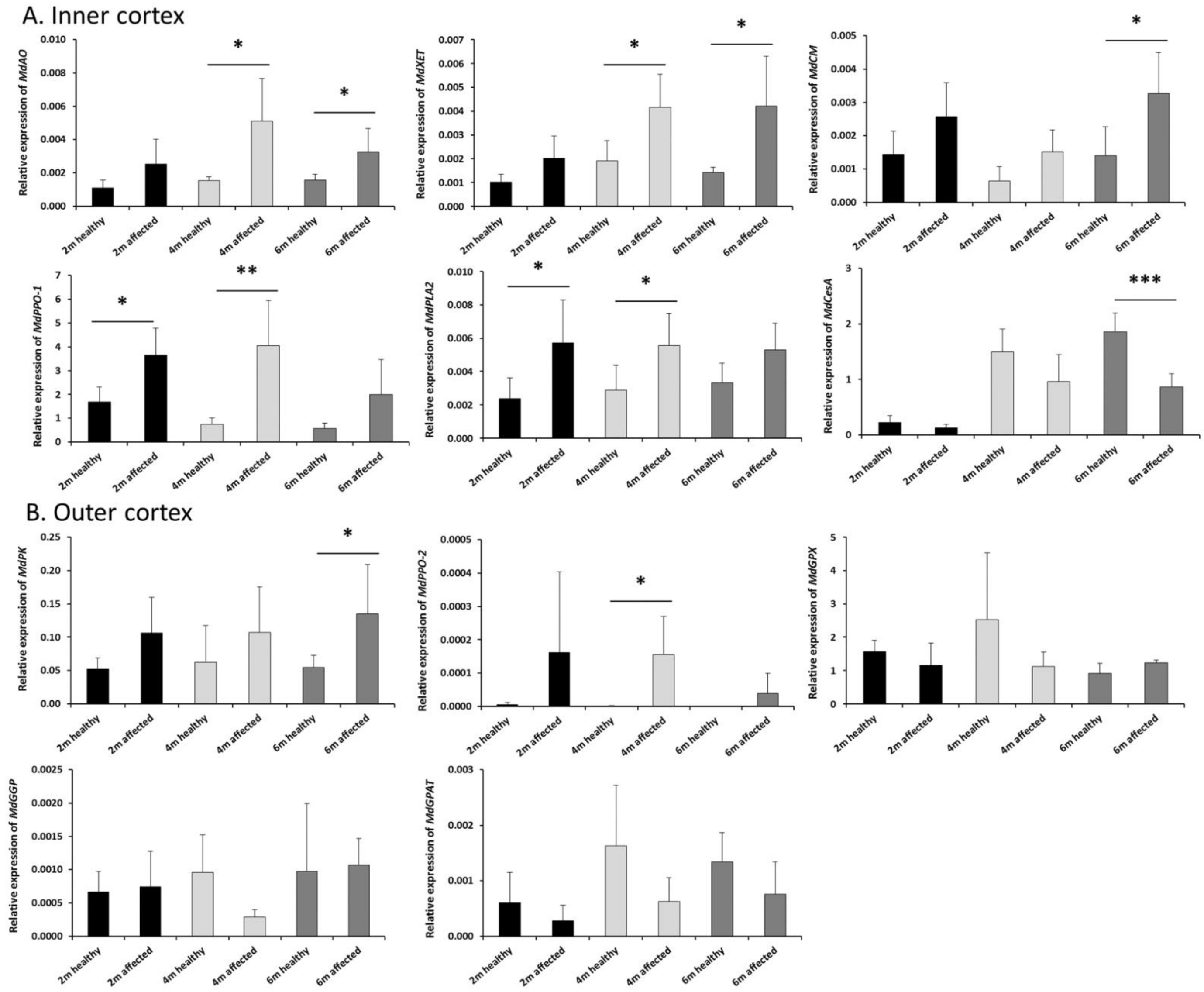
A. Inner cortex
Relative expression of MdAO
Relative expression of MdXET
Relative expression of MdCM
Relative expression of MdPPO-1
Relative expression of MdPLA2
Relative expression of MdCesA
B. Outer cortex
Relative expression of MdPK
Relative expression of MdPPO-2
Relative expression of MdGPX
Relative expression of MdGGP
Relative expression of MdGPAT
2m healthy
2m affected
4m healthy
4m affected
6m healthy
6m affected

Figure S4. Validation of RNA-Seq results by qRT-PCR. Relative expression of genes in 'Braeburn' apples stored for two, four, or six months under various CA conditions that were validated across one or two experiments. Inner cortex (A): ascorbate oxidase (*MdAO*; MDP0000610961), xyloglucan endotransglucosylase/hydrolase (*MdXET*; MDP0000873667), chorismate mutase (*MdCM*; MDP0000196554), polyphenol oxidase (*MdPPO-1*; MDP0000249183), phospholipase a2 (*MdPLA2*; MDP0000249250), and cellulose synthase (*MdCesA*; MDP0000289339). Outer cortex: pyruvate kinase (*MdPK*; MDP0000376244), polyphenol oxidase (*MdPPO-2*; MDP0000539552), glutathione peroxidase (*MdGPX*; MDP0000203927), L-galactose-1-phosphate phosphatase (*MdGPP*; MDP0000217438) and glycerol-3-phosphate acyltransferase (*MdGPAT*; MDP0000276629). qRT-PCR values were normalized against the geometrical mean of ubiquitin (MDP0000154072) and actin (MDP0000886327). The error bars represent standard deviation of five biological replications. Significance of the mean relative gene expression of each group was tested with Student's t-test (* $P<0.05$, ** $P<0.01$ and *** $P<0.001$) using SAS software.
