# Supplementary material for: Transcriptomic events associated with internal browning of apple during postharvest storage
Source: BMC Plant Biol. 2014 Nov 28;14:328. doi: 10.1186/s12870-014-0328-x (PMC4272543; doi:10.1186/s12870-014-0328-x)
Supplement: Additional file 2 — Table S1. Reads and their mapping on the Malus consensus sequences for inner and outer cortex. Table S2. Storage-related genes that were excluded from the analysis of RNA-Seq of browning disorder. Table S3. Differentially expressed genes between healthy and affected inner cortex. Table S4. Differentially expressed genes between healthy and affected outer cortex. Table S5. Primer sequences used for validation of RNA-Seq results by qRT-PCR. Table S6. Gene expression values (RPKM) of the different dehydroascorbate reductases (DHAR) as revealed by RNA-Seq. Table S7. The browning index (BI) of inner and outer cortex of apples used for RNA-Seq. Table S8. The browning index (BI) of inner and outer cortex of the validation set. [file 12870_2014_328_MOESM2_ESM.pdf]

Table S1. Reads and their mapping on the *Malus* consensus sequences for inner and outer cortex. Total reads are already filtered for quality as explained in methods. BI: browning index; H: healthy; A: affected.

Tissue	Condition	Sample	BI	Total reads	Mapped reads	% of reads mapped	Uniquely mapped reads	% of reads uniquely mapped	Expressed genes
**Inner cortex**	harvest	1	2.51	12,493,114	9,418,327	75.4	6,248,402	50	31,514
		2	2.69	18,023,367	13,444,539	74.6	8,923,539	49.6	31,057
		3	2.24	16,802,882	12,504,606	74.4	8,291,019	49.3	30,550
		4	2.25	13,669,513	10,125,981	74.1	6,735,742	49.2	30,574
	CA storage	H 1	3.09	14,549,147	10,567,214	72.6	6,999,393	48.1	31,762
		H 2	3.11	12,602,395	8,057,169	63.9	5,413,596	43	29,837
		H 3	3.59	13,091,620	9,315,803	71.2	6,145,770	46.9	31,918
		H 4	2.92	16,060,063	9,330,497	71.4	6,143,503	38.3	31,063
		A 1	4.20	10,264,536	5,616,180	54.7	3,752,090	36.6	31,396
		A 2	4.80	13,919,690	7,724,021	55.49	5,238,070	37.6	30,814
		A 3	4.73	15,233,638	11,162,657	73.3	7,388,822	48.5	31,553
		A 4	5.16	17,094,171	12,729,218	74.5	8,315,484	48.6	31,805
		A 5	4.57	16,189,945	10,046,655	62.1	6,612,379	40.8	29,756
		A 6	5.11	16,498,848	11,380,203	69	7,493,080	45.4	30,017
		A 7	4.99	12,969,157	9,110,696	70.3	6,037,323	46.6	31,650
		A 8	4.68	10,371,335	7,142,334	68.9	4,733,593	45.6	32,377
		A 9	4.13	16,682,327	11,760,062	70.5	7,684,353	46.1	31,639
		A 10	5.19	17,705,544	12,412,067	70.1	8,245,702	46.6	31,570
		A 11	4.86	18,563,457	13,870,779	74.7	8,954,873	48.2	31,371
		A 12	4.91	19,797,393	14,360,265	72.5	9,431,311	47.6	32,113
**Outer cortex**	harvest	1	2.60	24,484,547	18,536,811	75.7	12,250,575	50	30,984
		2	2.51	20,946,760	15,544,266	74.2	10,217,101	48.8	30,893
		3	2.39	23,211,304	17,422,124	75.1	11,423,322	49.2	30,291
		4	2.53	26,068,059	19,623,504	75.3	13,098,881	50.3	30,461
	CA storage	H 1	3.47	10,896,577	7,840,417	72	5,185,433	47.6	31,129
		H 2	3.44	10,136,941	7,253,958	71.6	4,836,958	47.7	30,028

Tissue	Condition	Sample	BI	Total reads	Mapped reads	% of reads mapped	Uniquely mapped reads	% of reads uniquely mapped	Expressed genes
		H 3	4.08	13,705,343	9,545,116	69.7	6,366,325	46.5	31,213
		H 4	3.89	10,339,407	7,168,360	69.3	4,769,320	46.1	29,916
		H 5	3.91	15,259,476	10,668,108	69.9	6,958,129	45.6	31,691
		H 6	3.70	16,110,944	11,224,925	69.7	7,424,481	46.1	31,347
		H 7	3.83	15,100,521	10,474,770	69.4	6,931,157	45.9	30,978
		H 8	3.60	15,097,671	9,915,539	65.7	6,579,666	43.6	24,261
		A 1	5.24	14,167,742	9,135,642	64.5	6,002,984	42.4	27,915
		A 2	4.72	13,939,890	9,971,066	71.5	6,572,052	47.2	30,079
		A 3	4.22	17,552,134	12,778,691	72.8	8,396,966	47.8	31,564
		A 4	4.94	14,738,221	10,547,795	71.6	6,956,115	47.2	31,241
		A 5	5.26	16,396,766	11,623,840	70.9	7,637,194	46.6	30,391
		A 6	4.61	14,409,797	10,212,375	70.9	6,686,750	46.4	31,376
		A 7	4.82	25,949,438	19,021,881	73.3	12,478,851	48.1	31,440
		A 8	4.65	20,527,076	14,803,284	72.1	9,776,704	47.6	31,094

Table S2. Storage-related genes that were excluded from the analysis of RNA-Seq of browning disorder. Differentially expressed genes between harvest and healthy apples stored for four months at CA conditions. (A) inner cortex, (B) outer cortex.

		RPKM		
**Malus ID**	**Description - Blast2Go**	**harvest**	**4 months healthy**	**p value**
**(A) inner cortex**				
MDP0000179769	protein chloroplastic-like	114.35	1.09	<0.0001
MDP0000238176	alpha-glucosidase -like	0.00	0.62	<0.0001
MDP0000191097	amino acid permease	8.69	23.67	<0.0001
MDP0000131308	fructokinase	5.80	48.59	<0.0001
MDP0000161521	Auxin-responsive protein	12.40	56.17	<0.0001
MDP0000233229	uncharacterized protein	7.71	31.86	<0.0001
MDP0000385086	uncharacterized protein	2.71	15.93	<0.0001
MDP0000127834	mitochondrial uncoupling protein 5-like	28.08	60.12	<0.0001
MDP0000141121	zinc finger an1 domain-containing stress-associated protein 12	3.09	7.30	<0.0001
MDP0000124013	probable chlorophyll b reductase chloroplastic-like	6.19	23.39	<0.0001
MDP0000229036	ethylene-responsive transcription factor abr1-like	0.27	13.09	<0.0001
MDP0000177898	tir-nbs-lrr resistance protein	0.06	0.45	<0.0001
MDP0000136685	trna pseudouridine synthase	1.08	0.13	<0.0001
MDP0000921224	ovate family protein	18.02	3.76	<0.0001
MDP0000138729	PAR1 protein	1.32	10.01	<0.0001
MDP0000686191	probable pectinesterase pectinesterase inhibitor 21-like	0.16	2.22	<0.0001
MDP0000885990	uncharacterized protein	802.61	1788.56	<0.0001
MDP0000442056	nbs-lrr resistance protein	0.19	10.03	<0.0001
MDP0000783676	probable sucrose-phosphate synthase 3-like	22.75	9.52	<0.0001
MDP0000303449	---NA---	0.34	19.05	<0.0001
MDP0000893203	abscisic insensitive 1b	1.31	3.73	<0.0001
MDP0000522996	tir-nbs-lrr resistance protein	0.16	0.92	<0.0001
MDP0000137919	ctp synthase	13.36	3.97	<0.0001
MDP0000761026	uncharacterized protein	12.08	42.43	<0.0001
MDP0000127807	GRAM domain	51.99	129.49	<0.0001
MDP0000238773	No apical meristem (NAM) protein	2.55	14.31	0.001
MDP0000231512	chromosome-associated kinesin kif4a-like	10.75	4.98	0.001
MDP0000120995	inactive rhomboid protein 1-like	36.83	79.14	0.001
MDP0000155158	rop guanine nucleotide exchange factor 1-like	0.00	0.77	0.001
MDP0000170739	C2H2-type zinc finger protein	0.13	3.51	0.001
MDP0000576110	---NA---	0.04	3.19	0.001
MDP0000242070	snap25 homologous protein snap33	10.40	21.85	0.001
MDP0000297722	protein phosphatase 2c	0.92	2.57	0.001
MDP0000853499	zinc finger an1 domain-containing stress-associated protein 12	3.67	7.46	0.001
MDP0000276665	alcohol dehydrogenase 1	3.11	13.97	0.001
MDP0000629000	calcium-dependent protein kinase 9-like	0.08	0.26	0.001
MDP0000382998	C2 calcium/lipid-binding plant phosphoribosyltransferase	8.44	16.89	0.002
MDP0000132011	receptor-like cytosolic serine threonine-protein kinase rbk2-like	0.06	0.27	0.002
MDP0000322543	senescence-inducible chloroplast stay-green protein	57.96	142.92	0.002
MDP0000284274	probable lrr receptor-like serine threonine-protein kinase at4g08850-like	0.09	0.46	0.002
MDP0000134666	pentatricopeptide repeat-containing protein	0.97	1.00	0.003
MDP0000321343	splicing factor u2af small subunit b	37.38	141.90	0.003
MDP0000183252	probable inactive purple acid phosphatase 27-like	0.02	0.36	0.003
MDP0000879957	---NA---	0.64	3.89	0.003
MDP0000155446	14 kda proline-rich protein	4.34	159.60	0.003
MDP0000295589	auxin-induced protein 22d-like	154.00	74.54	0.003
MDP0000168293	Laccase/Diphenol oxidase	0.08	7.26	0.004
MDP0000125954	receptor-like cytosolic serine threonine-protein kinase rbk2-like	0.11	0.76	0.004
MDP0000574166	alpha/beta-Hydrolases superfamily protein	0.94	3.21	0.004
MDP0000249986	60s ribosomal protein l6	0.20	0.82	0.004

		RPKM		
			4 months	
**Malus ID**	**Description - Blast2Go**	**harvest**	**healthy**	**p value**
MDP0000734582	uncharacterized protein	0.03	1.88	0.004
MDP0000263046	anaphase-promoting complex subunit cdc20-like	0.22	0.03	0.004
MDP0000213088	probable chlorophyll b reductase chloroplastic-like	0.36	3.82	0.005
MDP0000191851	chorismate mutase	0.20	2.03	0.005
MDP0000189486	protein phosphatase 2c	0.93	7.02	0.005
MDP0000257928	glutamine dumper-like	0.33	5.00	0.005
MDP0000283595	protein	0.54	1.01	0.005
MDP0000250895	o-fucosyltransferase family protein	0.94	2.21	0.007
MDP0000158089	beta subunit isoform 1	0.02	3.78	0.007
MDP0000319225	C2H2 Transcription Factor	0.17	11.08	0.008
MDP0000308990	dna topoisomerase 2-like	0.01	0.33	0.009
MDP0000119191	uncharacterized protein	0.09	0.74	0.009
MDP0000499104	nucleic acid binding related	7.04	3.43	0.009
MDP0000299445	ubiquitin-specific protease	0.12	0.96	0.010
MDP0000759010	pentatricopeptide repeat-containing protein chloroplastic-like	0.65	0.35	0.011
MDP0000212760	coatomer beta	0.00	4.67	0.011
MDP0000171708	protein notum homolog	0.98	8.06	0.012
MDP0000181436	coatomer beta	0.00	4.35	0.012
MDP0000165836	uncharacterized protein	0.93	1.25	0.013
MDP0000492906	serine threonine-protein kinase	0.02	1.61	0.013
MDP0000141888	Pentatricopeptide repeat	0.06	0.39	0.015
MDP0000131463	probable inactive purple acid phosphatase 1-like	0.17	0.58	0.015
MDP0000839736	coatomer beta	0.03	5.21	0.015
MDP0000368098	e3 ubiquitin-protein ligase ring1-like	6.03	15.30	0.016
MDP0000648621	---NA---	0.21	5.04	0.016
MDP0000215270	coatomer beta	0.04	3.92	0.018
MDP0000241976	abc transporter-like	0.02	0.35	0.019
MDP0000156103	myo-inositol oxygenase 1-like	0.11	0.37	0.020
MDP0000212606	g-type lectin s-receptor-like serine threonine-protein kinase b120-like	0.09	0.49	0.021
MDP0000232642	Translation initiation factor SUI1	2.48	6.84	0.021
MDP0000152448	coatomer subunit beta -1	0.02	2.98	0.021
MDP0000241227	phosphoribosyl pyrophosphate synthetase	0.54	0.20	0.022
MDP0000665063	ubiquitin-specific protease	0.01	0.09	0.023
MDP0000574087	cyclin-dependent kinase f-4-like	0.00	0.07	0.025
MDP0000763987	Metal-dependent protein hydrolase	0.62	0.23	0.025
MDP0000281865	serine threonine-protein kinase atr-like	0.29	0.43	0.026
MDP0000198624	probable receptor-like protein kinase at1g67000-like	0.51	0.99	0.028
MDP0000309287	disease resistance protein rga3-like	0.33	0.57	0.029
MDP0000124941	notchless protein homolog	0.29	0.78	0.032
MDP0000815242	uncharacterized protein	0.57	2.98	0.032
MDP0000319299	protein	1.17	1.65	0.032
MDP0000914171	uncharacterized protein	0.07	3.32	0.033
MDP0000359885	---NA---	0.00	1.43	0.034
MDP0000661404	Serine protease inhibitor (SERPIN)	0.02	0.13	0.035
MDP0000156208	peroxidase	0.01	0.14	0.036
MDP0000243237	peroxidase	0.02	0.06	0.036
MDP0000270571	transporter arsb-like	0.46	1.39	0.036
MDP0000330164	glutaredoxin	0.43	1.33	0.036
MDP0000311556	Zinc finger, DHHC-type	0.76	1.40	0.037
MDP0000329986	RHO protein GDP dissociation inhibitor	0.14	0.20	0.038
MDP0000897242	Pathogenesis-related thaumatin	0.16	0.09	0.038
MDP0000237597	phosphoglycerate mutase	0.71	0.15	0.039
MDP0000899392	xyloglucan galactosyltransferase katamari1-like	0.38	0.05	0.040
MDP0000563186	cyclic nucleotide-gated ion channel 1-like	0.16	0.51	0.040
MDP0000190918	proline-rich receptor-like protein kinase perk8-like	0.37	0.05	0.041
MDP0000386815	uncharacterized protein	0.03	0.13	0.044
MDP0000292097	receptor-like serine threonine kinase	0.02	0.31	0.046
MDP0000310071	histone chaperone asf1	0.08	0.05	0.046

		RPKM		
			4 months	
**Malus ID**	**Description - Blast2Go**	**harvest**	**healthy**	**p value**
MDP0000236662	cysteine proteinases-like protein	0.47	0.10	0.047
MDP0000308507	uncharacterized protein	0.19	0.42	0.048
MDP0000780462	S-adenosyl-L-methionine-dependent methyltransferase	0.32	0.49	0.048
MDP0000919183	nadh dehydrogenase-like	0.13	0.04	0.048
MDP0000301845	probable inorganic phosphate transporter 1-9-like	0.03	0.14	0.049
MDP0000527493	---NA---	0.26	0.39	0.049
MDP0000321182	g-type lectin s-receptor-like serine threonine-protein kinase at4g03230-like	0.34	0.46	0.050
MDP0000769609	---NA---	1.83	1.64	0.050
MDP0000922823	---NA---	0.15	0.04	0.050
MDP0000500882	protein kinase	0.03	0.24	0.050
MDP0000245681	esterase lipase domain-containing protein	0.99	0.61	0.050
MDP0000426742	mif4g domain-containing family protein	0.30	0.58	0.050
MDP0000301326	glucan endo- -beta-glucosidase 11-like	1.25	0.41	0.042
MDP0000223924	---NA---	0.04	0.55	0.048
MDP0000289075	ser thr protein kinase	0.13	0.25	0.049
MDP0000383458	bed zinc family dimerization isoform 1	0.55	0.96	0.049
**(B) outer cortex**				
MDP0000169913	protein	177.78	695.27	<0.0001
MDP0000346292	---NA---	55.54	201.13	<0.0001
MDP0000203199	---NA---	107.66	24.67	<0.0001
MDP0000290670	uncharacterized protein at2g23090	253.71	81.69	<0.0001
MDP0000299987	3-ketoacyl-acyl carrier protein synthase iii (kas iii)	0.35	0.00	<0.0001
MDP0000124187	zinc finger	0.51	0.04	<0.0001
MDP0000305017	f-box protein ore9	0.28	0.91	0.001
MDP0000951799	beta chain	17.26	5.29	0.001
MDP0000173666	protoporphyrinogen oxidase	9.43	4.65	0.001
MDP0000703622	phospholipid-transporting atpase 9-like	0.11	0.50	0.001
MDP0000611628	probable nadh dehydrogenase-like	2.88	0.33	0.001
MDP0000145885	vacuolar protein 8-like	0.59	0.07	0.002
MDP0000280025	ring u-box domain-containing protein	3.86	16.81	0.002
MDP0000247505	nudix hydrolase 2-like	0.66	0.04	0.002
MDP0000243873	bcl-2-associated athanogene 7	0.17	0.82	0.003
MDP0000527190	dormancy-associated mads-box transcription factor	0.32	0.04	0.003
MDP0000171517	transducin wd40 domain-containing protein	7.77	2.29	0.003
MDP0000292058	ferredoxin--nadp+ reductase	0.46	0.08	0.004
MDP0000219970	protein	0.06	0.82	0.004
MDP0000216136	dna-directed rna polymerase 3	0.36	1.01	0.005
MDP0000274867	cysteine synthase	12.49	32.10	0.005
MDP0000768772	ring zinc finger	18.89	25.62	0.006
MDP0000256359	pentatricopeptide repeat-containing protein	0.75	0.29	0.007
MDP0000255751	70-kd heat shock protein	2.38	5.26	0.007
MDP0000136240	---NA---	0.83	0.00	0.007
MDP0000146976	ubiquinol-cytochrome c reductase iron-sulfur subunit	0.18	0.66	0.008
MDP0000130405	fk506 binding	0.50	0.09	0.008
MDP0000253752	protein	0.38	1.02	0.009
MDP0000283898	dehydroquinate dehydratase shikimate dehydrogenase	0.39	0.99	0.009
MDP0000121990	flowering time control protein	0.25	0.87	0.009
MDP0000274635	f-box protein skip1	0.25	1.17	0.009
MDP0000506359	l-idonate 5-dehydrogenase	3.05	0.88	0.009
MDP0000303952	transcriptional coactivator-like protein	39.39	24.05	0.010
MDP0000293845	ankyrin repeat-containing protein at5g02620-like	0.37	0.77	0.011
MDP0000608906	rubber elongation factor	34.19	69.53	0.011
MDP0000157081	cc-nbs-lrr resistance protein	0.40	0.11	0.011
MDP0000733379	pentatricopeptide repeat-containing protein	1.50	0.57	0.013
MDP0000123719	protein	0.15	0.64	0.014
MDP0000259461	pentatricopeptide repeat-containing protein	0.24	0.78	0.016
MDP0000221643	alcohol dehydrogenase class iii	1.32	0.06	0.016
MDP0000260354	pentatricopeptide repeat-containing protein	0.19	0.57	0.018

		RPKM		
Malus ID	Description - Blast2Go	harvest	4 months healthy	p value
MDP0000314008	protein fizzy-related 3	0.16	0.63	0.018
MDP0000282034	phospholipid hydroperoxide glutathione peroxidase	0.34	0.87	0.018
MDP0000433058	protein	1.11	0.15	0.018
MDP0000179967	psi reaction center subunit iii	0.37	1.17	0.021
MDP0000541406	uncharacterized membrane protein at1g16860-like	0.07	0.55	0.022
MDP0000212753	vitamin-b12 independent methionine 5-methyltetrahydropteroyltriglutamate-homocysteine	0.80	0.23	0.023
MDP0000302106	myosin xi-2	0.16	0.63	0.024
MDP0000189320	l-ascorbate peroxidase	0.53	0.20	0.026
MDP0000280616	dehydration-responsive family protein	0.20	0.57	0.027
MDP0000941390	cytosolic delta subunit	0.36	0.88	0.028
MDP0000571954	d-3-phosphoglycerate chloroplastic-like	0.39	0.00	0.029
MDP0000203502	uncharacterized protein	1.79	8.71	0.031
MDP0000287729	udp-glucosyltransferase 74f2	0.02	0.87	0.032
MDP0000679950	uncharacterized protein loc100793942	0.07	0.73	0.033
MDP0000171451	pentatricopeptide repeat-containing protein	0.78	0.27	0.033
MDP0000764433	beta-amylase	0.12	0.47	0.034
MDP0000282855	protein	0.20	0.48	0.038
MDP0000679722	ubiquitin carboxyl-terminal hydrolase 12	0.11	0.65	0.040
MDP0000125338	probable disease resistance protein at5g63020-like	0.42	1.09	0.041
MDP0000126388	pentatricopeptide repeat-containing protein	0.35	0.62	0.041
MDP0000579770	at1g62520-like protein	0.23	0.06	0.041
MDP0000563592	somatic embryogenesis receptor kinase 1	0.19	0.70	0.041
MDP0000402954	ubiquitin conjugating enzyme	0.50	0.84	0.041
MDP0000778465	squamosa promoter-binding-like protein 6-like	0.43	0.19	0.041
MDP0000174971	cellulose synthase	0.07	0.51	0.041
MDP0000213015	cell division cycle 5-like	0.57	0.20	0.042
MDP0000267822	ethylene-regulated transcript 2	0.17	0.54	0.043
MDP0000706020	bifunctional dihydroflavonol 4-reductase flavanone 4-reductase-like	0.26	1.83	0.043
MDP0000280642	disease resistance rpp13-like protein 1-like	0.18	0.63	0.044
MDP0000267154	methyltransferase-like protein 1-like	0.47	0.21	0.044
MDP0000357461	centromere-specific h3 variant protein	0.55	0.81	0.045
MDP0000736915	tir-nbs-lrr class disease resistance protein	0.37	0.60	0.046
MDP0000279863	ubiquitin-associated ts-n domain-containing protein	0.50	0.84	0.046
MDP0000144874	nbs-containing resistance-like protein	0.40	0.24	0.046
MDP0000230621	callose synthase	1.07	0.44	0.048
MDP0000147704	tir-nbs-lrr rct1-like resistance protein	0.67	0.22	0.048
MDP0000468411	ataf2 protein	0.11	0.73	0.049
MDP0000579628	f-box kelch-repeat protein	0.07	1.11	0.049
MDP0000183138	protein	0.40	0.89	0.049
MDP0000149857	armadillo beta-catenin repeat family protein	0.06	0.13	0.049
MDP0000299698	probable receptor-like protein kinase at5g56460-like	0.72	0.53	0.049
MDP0000199093	f-box protein at1g67340-like	0.08	0.19	0.049
MDP0000210612	mitogen-activated protein kinase kinase 6-like	0.68	0.48	0.049
MDP0000226270	isoflavone 2 -hydroxylase-like	0.00	0.12	0.049
MDP0000127832	hypothetical protein VITISV_037618 [Vitis vinifera]	0.44	0.23	0.049
MDP0000555634	uncharacterized protein	0.62	0.86	0.049
MDP0000352480	---NA---	0.37	0.61	0.049
MDP0000821908	nuclear transcription factor y subunit b-5-like	0.18	0.42	0.049
MDP0000205356	lrr receptor-like serine threonine-protein kinase gso2-like	0.38	0.57	0.049
MDP0000585979	tvp38 tmem64 family membrane protein slr0305-like	0.85	0.42	0.050
MDP0000775087	protein	0.99	0.60	0.050
MDP0000828703	actin-related protein 4-like	1.34	0.94	0.050
MDP0000181302	protein ilityhia	0.59	0.30	0.050
MDP0000341885	uncharacterized protein	0.10	0.49	0.050
MDP0000179365	monocopper oxidase-like protein sku5-like	1.39	0.91	0.050
MDP0000223843	long-chain acyl- synthetase	0.44	0.72	0.050
MDP0000229508	isoflavone 2 -hydroxylase-like	0.00	0.12	0.050

		RPKM		
**Malus ID**	**Description - Blast2Go**	**harvest**	**4 months healthy**	**p value**
MDP0000897919	---NA---	0.93	0.28	0.050
MDP0000268973	protein	1.75	0.73	0.050
MDP0000219586	structural maintenance of chromosomes 1	0.43	1.44	0.050

Table S3. Differentially expressed genes between healthy and affected inner cortex. Apples were stored for four months at controlled atmosphere conditions. P-values for the differentially expressed genes were calculated using PLS-DA analysis performed in The Unscrambler, where gene expression values (RPKM) was used as predictor variables and the two class distinctions (healthy–affected) as response variables.

			RPKM		
**Malus ID**	**Description - Blast2Go**	**p value**	**healthy**	**affected**	**fold change**
**(A) induced in affected apples**					
MDP0000904458	fasciclin-like arabinogalactan	<0.0001	193.78	525.70	2.71
MDP0000192364	pyruvate dehydrogenase e1 component subunit alpha-like	<0.0001	14.53	29.39	2.02
MDP0000723275	probable arabinose 5-phosphate isomerase-like	<0.0001	18.80	32.91	1.75
MDP0000827881	---NA---	<0.0001	852.51	2322.54	2.72
MDP0000283158	alpha beta-hydrolase domain-containing protein, lipase	<0.0001	4.51	7.92	1.76
MDP0000274120	gras family transcription factor	<0.0001	10.53	16.68	1.58
MDP0000275058	subtilisin-like protease	<0.0001	4.47	14.79	3.31
MDP0000145463	exocyst subunit exo70 family protein H7	<0.0001	10.39	26.98	2.60
MDP0000295823	probable lactate/malate dehydrogenase	<0.0001	20.06	30.24	1.51
MDP0000610961	l-ascorbate oxidase homolog	<0.0001	30.35	132.43	4.36
MDP0000350457	uncharacterized protein	<0.0001	6.51	16.45	2.53
MDP0000793014	phospho-n-acetylmuramoyl-pentapeptide-transferase homolog	0.0001	10.28	20.09	1.95
MDP0000165526	protein kinase	0.0001	51.45	82.13	1.60
MDP0000873667	xyloglucan endotransglucosylase/hydrolase	0.0001	52.59	229.40	4.36
MDP0000252195	ferredoxin-thioredoxin reductase	0.0001	10.84	16.70	1.54
MDP0000145512	protein yipf5-like	0.0001	70.79	108.55	1.53
MDP0000152807	nbs-lrr-like protein	0.0002	5.76	9.46	1.64
MDP0000324681	---NA---	0.0002	636.07	1445.08	2.27
MDP0000303737	50s ribosomal protein l3-2	0.0002	3.53	6.53	1.85
MDP0000185643	probable galacturonosyltransferase-like 9-like	0.0002	10.62	20.35	1.92
MDP0000321326	glycosyl hydrolase family 17 family protein	0.0002	0.55	3.25	5.90
MDP0000206680	reticuline oxidase-like	0.0003	1.48	5.13	3.45
MDP0000433544	NAD-dependent epimerase/dehydratase	0.0003	4.69	10.36	2.21
MDP0000127240	cell division cycle protein 27 homolog b-like isoform x1	0.0003	0.28	1.09	3.86
MDP0000623212	heat shock protein	0.0004	8.85	28.34	3.20
MDP0000125377	methyltransferase, MT-A70	0.0004	0.21	1.23	5.74
MDP0000846032	chlorophyll a b binding protein	0.0004	7.10	11.74	1.65
MDP0000235663	probable glucan endo- -beta-glucosidase a6-like	0.0004	2.97	6.43	2.16
MDP0000371724	aldose 1-epimerase	0.0004	18.76	29.68	1.58
MDP0000196554	chorismate mutase/APX	0.0005	11.63	19.96	1.72
MDP0000249183	polyphenol oxidase	0.0005	1767.32	3674.83	2.08
MDP0000254930	desumoylating isopeptidase 1-like	0.0006	3.56	7.29	2.05
MDP0000126808	abscisic acid (aba)-deficient 4	0.0006	31.05	76.31	2.46
MDP0000332125	patellin-6-like	0.0007	5.78	22.58	3.91
MDP0000241183	uncharacterized protein	0.0007	1.80	5.30	2.94
MDP0000615196	glutaredoxin family protein	0.0008	0.32	1.18	3.64
MDP0000260834	calcium-dependent protein kinase	0.0010	25.34	38.49	1.52
MDP0000702557	UDP-glucuronosyl/UDP-glucosyltransferase	0.0010	30.37	48.24	1.59
MDP0000385309	prenylated rab receptor 2	0.0013	47.96	76.96	1.60
MDP0000260512	4-coumarate: ligase	0.0013	0.16	1.05	6.71
MDP0000275800	transcription factor myb44-like	0.0015	20.37	40.20	1.97
MDP0000148817	ef-hand calcium binding	0.0017	38.17	58.04	1.52
MDP0000139849	e3 ubiquitin-protein ligase rhf2a-like	0.0017	2.39	6.74	2.82
MDP0000283975	protein kinase	0.0017	1.72	2.67	1.55
MDP0000509768	two-component response regulator	0.0018	4.97	8.64	1.74
MDP0000921834	actin	0.0018	7.69	15.54	2.02
MDP0000951463	---NA---	0.0019	191.32	434.42	2.27
MDP0000132630	arginyl-trna synthetase	0.0019	18.59	32.60	1.75
MDP0000213911	26s proteasome regulatory particle non-atpase subunit	0.0021	7.14	12.13	1.70
MDP0000272599	Ribosomal protein L18	0.0024	28.80	46.00	1.60
MDP0000120022	acid phosphatase 1	0.0024	22.91	35.55	1.55
MDP0000915501	salt tolerance homolog2	0.0029	3.03	4.78	1.58
MDP0000194823	Calcium-dependent lipid-binding protein	0.0030	11.66	30.24	2.59
MDP0000270747	uncharacterized protein	0.0031	33.93	56.63	1.67
MDP0000794484	alpha/beta-Hydrolases	0.0033	2.60	6.33	2.43
MDP0000249250	phospholipase a2	0.0037	0.49	4.25	8.65
MDP0000146621	nadh-cytochrome b5 reductase-like protein	0.0038	4.39	8.03	1.83

Malus ID	Description - Blast2Go	p value	RPKM healthy	RPKM affected	fold change
MDP0000177079	beta-glucosidase	0.0038	5.18	17.71	3.42
MDP0000309977	acyl-coenzyme a thioesterase 13-like	0.0040	0.95	2.23	2.36
MDP0000725969	phosphoglycolate phosphatase	0.0043	5.11	7.71	1.51
MDP0000820365	prenylated rab acceptor	0.0043	46.57	91.23	1.96
MDP0000947222	gcn5-related n-acetyltransferase family protein	0.0056	3.32	7.59	2.29
MDP0000349671	---NA---	0.0062	11.26	19.03	1.69
MDP0000170414	flavin-containing monooxygenase	0.0065	0.67	2.98	4.44
MDP0000119813	cysteine protease family protein	0.0069	1.73	5.13	2.97
MDP0000261625	polyamine oxidase	0.0071	1.10	3.03	2.75
MDP0000946378	Protein of unknown function DUF580	0.0074	1.56	3.82	2.45
MDP0000320551	dtw domain-containing protein	0.0079	4.69	8.19	1.75
MDP0000305592	dynamin-related protein	0.0080	0.15	1.05	6.82
MDP0000849585	alpha/beta-Hydrolase	0.0085	2.85	5.69	2.00
MDP0000181718	---NA---	0.0087	0.35	2.86	8.18
MDP0000124719	zinc finger family protein	0.0087	5.29	9.43	1.78
MDP0000186495	rop guanine nucleotide exchange factor 1-like	0.0088	0.57	1.58	2.77
MDP0000769596	uncharacterized protein	0.0088	3.98	16.94	4.26
MDP0000905600	chaperonin-like protein isoform 2	0.0095	1.47	5.26	3.57
MDP0000576682	butyrate-- ligase peroxisomal-like	0.0095	0.20	1.30	6.63
MDP0000209470	deoxyribodipyrimidine photo-lyase-like	0.0100	0.83	1.81	2.17
MDP0000153857	disease resistance family protein / LRR family protein	0.0100	0.50	1.04	2.07
MDP0000807958	EXORDIUM like 3	0.0110	25.29	40.01	1.58
MDP0000264673	---NA---	0.0117	1.68	4.74	2.82
MDP0000484742	calcium-transporting atpase plasma membrane-type-like	0.0118	0.13	1.06	8.31
MDP0000320189	Transcriptional coactivator-pterin dehydratase	0.0124	69.26	111.00	1.60
MDP0000722904	cyclin-dependent kinase	0.0125	5.77	12.29	2.13
MDP0000298312	nuclear transport factor 2 -like protein	0.0136	1.28	2.05	1.60
MDP0000248714	Phytanoyl-CoA dioxygenase	0.0143	3.33	5.02	1.50
MDP0000142665	uncharacterized protein	0.0144	17.95	29.03	1.62
MDP0000269554	gpi ethanolamine phosphate transferase 1-like	0.0150	32.28	54.16	1.68
MDP0000129305	probable phosphoglycerate mutase -like	0.0155	0.91	1.80	1.98
MDP0000378642	eukaryotic translation initiation factor 4e	0.0173	18.42	34.94	1.90
MDP0000803089	pentatricopeptide repeat-containing protein	0.0180	3.99	7.44	1.87
MDP0000833444	diacylglycerol kinase-like	0.0181	2.14	5.15	2.41
MDP0000251669	thioredoxin	0.0190	18.00	28.15	1.56
MDP0000505812	prenylated RAB acceptor 1.E	0.0198	16.42	26.15	1.59
MDP0000334368	histone h2a	0.0206	14.56	23.09	1.59
MDP0000282670	probable pyridoxal biosynthesis protein pdx1-like	0.0223	0.80	2.54	3.17
MDP0000127549	3-hexulose-6-phosphate isomerase-like	0.0224	1.48	3.67	2.48
MDP0000219091	FAD/NAD(P)-binding oxidoreductase	0.0228	17.88	31.09	1.74
MDP0000410930	RNA-binding (RRM/RBD/RNP motifs) protein	0.0230	0.52	1.02	1.96
MDP0000231671	serine threonine-protein	0.0236	0.47	1.05	2.23
MDP0000720268	---NA---	0.0249	1.12	5.77	5.16
MDP0000376617	calmodulin-binding protein	0.0251	3.00	5.39	1.80
MDP0000148886	uncharacterized protein	0.0279	2.63	4.13	1.57
MDP0000201862	embryogenesis-associated protein emb8-like	0.0320	20.10	35.89	1.79
MDP0000201047	invertase inhibitor-like	0.0325	0.96	3.70	3.87
MDP0000616781	copper transporter	0.0326	0.67	2.17	3.22
MDP0000838746	transmembrane emp24 domain-containing protein p24delta3-like	0.0330	3.68	8.26	2.24
MDP0000233778	thylakoid lumen protein	0.0344	0.41	1.59	3.89
MDP0000199767	Rho GTPase activation protein	0.0348	3.86	7.46	1.93
MDP0000523159	uncharacterized protein	0.0353	1.52	4.12	2.71
MDP0000378275	uncharacterized n-acetyltransferase ycf52-like	0.0353	6.76	10.81	1.60
MDP0000795736	ferredoxin	0.0361	8.12	12.26	1.51
MDP0000180011	Thioesterase/thiol ester dehydrase-isomerase	0.0367	3.97	6.02	1.52
MDP0000822876	beta- -mannosyl-glycoprotein 4-beta-n-acetylglucosaminyltransferase-like	0.0368	4.38	10.07	2.30
MDP0000321774	chaperone protein chloroplastic-like	0.0410	20.67	31.19	1.51
MDP0000700383	kda class ii heat shock protein	0.0417	1.44	3.65	2.53
MDP0000267416	nucleoporin autopeptidase	0.0418	5.94	10.53	1.77
MDP0000119714	outer envelope protein of 80 kDa	0.0448	1.29	1.98	1.53
MDP0000162220	uncharacterized protein	0.0458	6.02	10.09	1.68
MDP0000174556	60s ribosomal protein l38-like	0.0468	1.63	2.52	1.54
MDP0000124428	rcc1 and btb domain-containing protein	0.0471	0.88	2.11	2.38
MDP0000768772	ring zinc finger	0.0477	34.57	52.25	1.51
MDP0000406918	apyrase-like protein	0.0493	3.95	8.53	2.16

(B) repressed in affected apples

			RPKM		
Malus ID	Description - Blast2Go	p value	healthy	affected	fold change
MDP0000282938	lysine-specific demethylase 3a-b	<0.0001	3.64	2.35	1.55
MDP0000200978	probable lrr receptor-like serine threonine-protein kinase at3g47570-like	<0.0001	2.48	0.62	4.01
MDP0000223159	rhomboid-like protein 15	<0.0001	16.97	11.16	1.52
MDP0000148287	serine threonine-protein kinase pbs1	0.0002	18.90	11.39	1.66
MDP0000642530	pentatricopeptide repeat-containing protein at1g71490-like	0.0002	7.49	4.93	1.52
MDP0000252413	soluble starch synthase chloroplastic amyloplastic-like	0.0002	6.58	3.82	1.72
MDP0000132594	uncharacterized protein	0.0002	6.32	3.57	1.77
MDP0000308305	probable lrr receptor-like serine threonine-protein kinase at3g47570-like	0.0004	1.55	0.45	3.41
MDP0000283164	pseudouridine-5 -monophosphatase-like	0.0004	36.50	21.42	1.70
MDP0000195070	probable lrr receptor-like serine threonine-protein kinase at1g07650-like	0.0004	15.44	10.11	1.53
MDP0000146856	erythronate-4-phosphate dehydrogenase family protein isoform 1	0.0007	21.47	11.97	1.79
MDP0000307943	transcription factor hbp-1b -like	0.0008	1.28	0.22	5.87
MDP0000677129	atp binding	0.0008	18.22	10.69	1.70
MDP0000575170	organic cation carnitine transporter 4-like	0.0009	71.77	47.23	1.52
MDP0000155229	gibberellin 2-beta-dioxygenase 8-like	0.0009	3.51	1.06	3.31
MDP0000214924	sodium hydrogen exchanger 7-like	0.0012	29.29	17.52	1.67
MDP0000585464	rna polymerase ii c-terminal domain phosphatase-like 4-like isoform x2	0.0012	7.41	3.22	2.30
MDP0000842746	tmv resistance protein n-like	0.0021	5.71	2.25	2.54
MDP0000257308	abc transporter c family member 10-like	0.0023	14.03	9.14	1.54
MDP0000753418	mediator of rna polymerase ii transcription subunit 33a-like	0.0023	3.72	2.33	1.60
MDP0000077460	NAD(P)-binding-cinnamyl alcohol dehydrogenase 2	0.0026	25.03	15.20	1.65
MDP0000251447	uncharacterized protein loc100816823	0.0027	9.07	4.32	2.10
MDP0000309120	alpha beta-hydrolases superfamily protein	0.0028	26.01	16.21	1.60
MDP0000222416	rna polymerase ii transcription mediator	0.0029	3.46	1.83	1.90
MDP0000464276	calcineurin b-like protein 10-like	0.0030	17.03	4.34	3.92
MDP0000138296	uncharacterized protein	0.0034	23.58	15.21	1.55
MDP0000134193	at3g58030-like protein	0.0036	11.78	7.68	1.53
MDP0000622920	phosphoenolpyruvate carboxylase	0.0036	8.35	5.47	1.53
MDP0000181414	alpha-ketoglutarate-dependent dioxygenase alkb-like protein	0.0045	6.69	4.16	1.61
MDP0000234292	cytochrome p450 86b1-like	0.0045	1.75	0.58	3.02
MDP0000532490	probable peptide nitrate transporter at5g14940-like	0.0046	2.35	0.76	3.11
MDP0000860957	tmv resistance protein n-like	0.0046	1.51	0.87	1.72
MDP0000290727	---NA---	0.0046	1.46	0.39	3.73
MDP0000793733	uncharacterized protein	0.0050	17.87	10.25	1.74
MDP0000366725	---NA---	0.0051	2.72	1.37	1.99
MDP0000288494	protection of telomeres 1 protein	0.0053	2.44	1.34	1.82
MDP0000201288	succinate dehydrogenase 5	0.0053	1.08	0.26	4.17
MDP0000349941	short-chain dehydrogenase tic chloroplastic-like	0.0056	2.44	1.03	2.36
MDP0000158403	pre-rrna-processing protein tsr1 homolog	0.0065	17.29	11.35	1.52
MDP0000137590	zinc finger ccch domain-containing protein 64-like	0.0066	4.09	2.19	1.87
MDP0000313910	btb poz domain-containing protein at3g05675-like isoform x1	0.0073	5.99	3.96	1.51
MDP0000640815	kh domain-containing protein at4g18375-like	0.0075	7.63	4.26	1.79
MDP0000258518	adp-glucose pyrophosphorylase large subunit 2 family protein	0.0076	1.63	0.77	2.12
MDP0000451182	peroxidase 66-like	0.0087	1.12	0.24	4.62
MDP0000260604	nitrate transporter -like	0.0090	2.19	1.02	2.14
MDP0000628075	uncharacterized methyltransferase chloroplastic-like	0.0094	2.77	1.16	2.40
MDP0000179232	u-box domain-containing protein 9	0.0096	13.62	9.07	1.50
MDP0000218942	uncharacterized protein	0.0096	2.47	1.57	1.57
MDP0000334744	---NA---	0.0100	1.53	0.28	5.46
MDP0000358111	---NA---	0.0102	2.41	0.47	5.17
MDP0000162193	lysine-specific histone demethylase-like protein	0.0103	6.59	4.02	1.64
MDP0000616949	probable pectinesterase pectinesterase inhibitor 6-like	0.0104	1.04	0.44	2.37
MDP0000183785	protection of telomeres 1 protein	0.0107	2.14	1.24	1.72
MDP0000824067	probable calcium-binding protein cml49-like	0.0108	31.17	20.48	1.52
MDP0000148800	tmv resistance protein n-like	0.0114	1.24	0.50	2.47
MDP0000322059	probable inactive leucine-rich repeat receptor-like protein kinase at3g03770-like	0.0116	6.32	4.11	1.54
MDP0000174803	dna-directed rna polymerase subunit	0.0117	1.24	0.56	2.24
MDP0000281875	transcription initiation factor tfiih subunit h4	0.0123	10.78	6.77	1.59
MDP0000195381	uncharacterized protein	0.0127	4.18	2.52	1.66
MDP0000289339	cellulose synthase-like protein h1-like	0.0129	1.90	1.03	1.85
MDP0000077753	elongation factor	0.0135	105.15	69.57	1.51
MDP0000798440	cytochrome c biogenesis fn	0.0136	2.67	1.52	1.76

Malus ID	Description - Blast2Go	p value	RPKM healthy	RPKM affected	fold change
MDP0000142433	uncharacterized aarf domain-containing protein kinase chloroplastic-like	0.0141	5.79	3.58	1.62
MDP0000121968	tmv resistance protein n-like	0.0149	2.17	1.05	2.06
MDP0000213491	isoleucyl-trna synthetase	0.0153	1.32	0.77	1.71
MDP0000136501	phosphatidylinositol glycan anchor biosynthesis class u	0.0157	31.95	20.52	1.56
MDP0000355568	---NA---	0.0160	3.09	0.47	6.55
MDP0000198410	aconitate hydratase 1	0.0165	19.75	11.72	1.69
MDP0000534408	spindle assembly checkpoint component	0.0165	6.10	3.40	1.80
MDP0000573982	rna polymerase-associated protein leo1-like	0.0171	72.47	45.41	1.60
MDP0000864525	zinc transporter at3g08650-like	0.0173	4.56	3.00	1.52
MDP0000299891	uncharacterized protein	0.0184	8.72	5.76	1.51
MDP0000245414	---NA---	0.0187	2.22	0.69	3.21
MDP0000250359	protein tyrosine expressed	0.0200	6.90	3.66	1.89
MDP0000928700	peptidyl-prolyl cis-trans isomerase pasticcino1-like	0.0203	29.65	19.14	1.55
MDP0000400817	ctl-like protein ddb_g0274487-like	0.0217	2.33	0.97	2.39
MDP0000277827	pantothenate kinase 2-like	0.0226	2.44	1.23	1.99
MDP0000104520	eukaryotic translation elongation factor 1b alpha-subunit	0.0232	2.85	0.47	6.05
MDP0000134027	protein kinase chloroplastic-like	0.0240	59.07	38.45	1.54
MDP0000290600	uncharacterized protein	0.0254	1.13	0.44	2.56
MDP0000845124	prenyl-dependent caax	0.0255	1.82	0.59	3.10
MDP0000254437	uncharacterized protein	0.0262	6.82	3.80	1.79
MDP0000198630	uncharacterized protein	0.0262	5.58	3.27	1.71
MDP0000242009	shatterproof-like protein	0.0267	14.79	8.32	1.78
MDP0000886424	peptide nitrate transporter	0.0268	7.15	4.04	1.77
MDP0000214084	wd repeat-containing protein 26-like	0.0269	4.92	2.58	1.91
MDP0000568065	amidohydrolase -like	0.0283	2.95	1.66	1.77
MDP0000497333	probable lrr receptor-like serine threonine-protein kinase at4g08850-like	0.0289	4.45	2.26	1.97
MDP0000248462	carbamoyl-phosphate synthase large chain-like	0.0289	2.02	1.19	1.70
MDP0000135634	magnesium transporter nipa2-like	0.0293	7.89	4.89	1.61
MDP0000243444	trna (guanine-n -)-methyltransferase-like	0.0298	7.85	2.43	3.23
MDP0000753976	uncharacterized fcp1 homology domain-containing	0.0299	1.87	0.65	2.86
MDP0000156108	Protein kinase	0.0307	2.83	1.55	1.83
MDP0000137283	protein	0.0314	6.71	4.32	1.55
MDP0000200896	1-aminocyclopropane-1-carboxylate oxidase	0.0319	74.26	39.10	1.90
MDP0000173152	mrna turnover protein 4	0.0323	1.70	1.07	1.59
MDP0000817651	mitochondrial substrate carrier family protein	0.0338	7.06	3.40	2.07
MDP0000850418	pentatricopeptide repeat-containing protein mitochondrial-like	0.0358	1.66	0.84	1.98
MDP0000836165	probable pectinesterase pectinesterase inhibitor 41-like	0.0363	2.42	1.21	2.00
MDP0000314385	uncharacterized protein	0.0371	6.70	3.78	1.77
MDP0000296941	tmv resistance protein n-like	0.0372	5.42	2.95	1.84
MDP0000266556	disease resistance rpp13-like protein 1-like	0.0379	4.89	2.71	1.81
MDP0000654455	---NA---	0.0379	16.18	8.30	1.95
MDP0000273425	diacylglycerol kinase-like	0.0396	4.44	2.73	1.63
MDP0000252113	probable disease resistance protein at5g66900-like	0.0410	3.35	1.65	2.03
MDP0000520089	desacetoxyvindoline 4-	0.0412	5.41	3.53	1.53
MDP0000126680	tyrosine specific protein phosphatase family protein	0.0422	36.20	21.12	1.71
MDP0000311330	protease do-like 7-like	0.0425	1.24	0.32	3.81
MDP0000298810	---NA---	0.0455	3.68	1.52	2.42
MDP0000316241	patellin-6-like	0.0470	2.80	1.10	2.55
MDP0000823574	dna binding	0.0481	9.23	6.03	1.53
MDP0000207408	nac domain-containing protein 7-like	0.0490	11.44	6.04	1.89

Table S4. Differentially expressed genes between healthy and affected outer cortex. Apples were stored for four months at controlled atmosphere conditions. P-values for the differentially expressed genes were calculated using PLS-DA analysis performed in The Unscrambler, where gene expression values (RPKM) was used as predictor variables and the two class distinctions (healthy – affected) as response variables.

			RPKM		
**Malus ID**	**Description - Blast2Go**	**p value**	**healthy**	**affected**	**fold change**
**(A) induced in affected apples**					
MDP0000367345	peptide methionine sulfoxide reductase-like	<0.0001	24.18	37.89	1.57
MDP0000305007	pyridoxal phosphate-dependent transferases superfamily protein isoform 1	<0.0001	6.12	9.35	1.53
MDP0000349972	hypothetical protein CICLE_v10007195mg	<0.0001	7.71	17.52	2.27
MDP0000195207	fad nad -binding oxidoreductase domain-containing protein	<0.0001	2.48	4.09	1.65
MDP0000337239	---NA---	<0.0001	11.66	28.93	2.48
MDP0000265369	proteasome maturation factor ump1 family protein	<0.0001	16.88	25.44	1.51
MDP0000175531	ac012654_15est gb	<0.0001	17.11	26.43	1.54
MDP0000252754	calcium-transporting atpase plasma membrane-type-like	<0.0001	1.62	3.47	2.15
MDP0000786461	low temprature induced-like protein	<0.0001	776.83	1240.68	1.60
MDP0000245732	60s ribosomal protein l35a-1-like	<0.0001	19.10	29.10	1.52
MDP0000250525	peptidyl-trna hydrolase ptrhd1-like	0.0006	20.11	30.31	1.51
MDP0000135887	ubiquitin-fold modifier-conjugating enzyme	0.0006	61.98	112.95	1.82
MDP0000278185	copper-transporting atpase paa1	0.0006	4.59	8.06	1.76
MDP0000296383	Pentatricopeptide repeat	0.0007	1.80	3.10	1.72
MDP0000234503	sac3 ganp nin1 mts3 eif-3 p25 isoform 1	0.0008	1.61	2.51	1.56
MDP0000889819	copper transporter	0.0008	151.39	240.40	1.59
MDP0000307742	late embryogenesis abundant hydroxyproline-rich glycoprotein	0.0008	15.67	24.37	1.56
MDP0000314866	rrna-processing protein fcf2-like	0.0008	1.58	3.57	2.27
MDP0000208202	wd repeat domain phosphoinositide-interacting protein 3-like	0.0009	5.50	8.77	1.59
MDP0000126272	f-box protein skip22-like	0.0010	0.99	3.43	3.48
MDP0000378642	eukaryotic translation initiation factor 4e	0.0011	22.54	38.91	1.73
MDP0000232454	transcription factor myb44-like	0.0012	19.35	29.78	1.54
MDP0000205579	polyketide cyclase dehydrase and lipid transport superfamily protein	0.0012	3.97	5.98	1.51
MDP0000539579	---NA---	0.0012	43.95	69.70	1.59
MDP0000435937	cation efflux family protein	0.0012	22.03	34.52	1.57
MDP0000676812	hypothetical protein PRUPE_ppa017894mg	0.0014	6.57	15.49	2.36
MDP0000288212	protease 4-like	0.0014	7.91	13.08	1.65
MDP0000278819	hypothetical protein PRUPE_ppa026893mg, partial	0.0015	4.03	7.33	1.82
MDP0000292247	60s ribosomal protein l36-2-like	0.0015	24.74	37.78	1.53
MDP0000416466	universal stress protein family protein	0.0016	5.48	12.96	2.36
MDP0000271021	protease do-like 7-like	0.0019	8.34	14.41	1.73
MDP0000270312	Neutral/alkaline non-lysosomal ceramidase	0.0019	2.81	4.71	1.68
MDP0000598064	hd domain class transcription factor	0.0019	0.47	1.12	2.39
MDP0000084546	cycloartenol synthase	0.0020	1.47	2.21	1.51
MDP0000164400	60s acidic ribosomal protein p0	0.0021	1.40	3.03	2.17
MDP0000269612	cinnamoyl- reductase family protein	0.0023	210.28	315.08	1.50
MDP0000187983	uncharacterized methyltransferase chloroplastic-like	0.0023	0.43	1.50	3.45
MDP0000153843	upf0202 protein at1g10490-like	0.0024	0.48	1.36	2.82
MDP0000366847	hypothetical protein PRUPE_ppa013118mg	0.0024	25.59	39.16	1.53
MDP0000411897	pleckstrin homology domain-containing protein 1	0.0024	25.26	37.99	1.50
MDP0000356032	---NA---	0.0025	2.01	4.71	2.34
MDP0000251581	succinate dehydrogenase	0.0026	25.49	42.53	1.67
MDP0000248806	probable plastid-lipid-associated protein chloroplastic-like	0.0028	8.14	13.03	1.60
MDP0000285549	protein-methionine-s-oxide reductase	0.0028	4.95	11.60	2.34
MDP0000245809	uncharacterized loc101221865	0.0029	11.62	18.30	1.57
MDP0000312567	transmembrane emp24 domain-containing protein p24delta9-like	0.0030	1.88	5.10	2.71
MDP0000322999	---NA---	0.0030	1.72	7.68	4.48
MDP0000242491	salicylic acid-binding protein 2-like	0.0031	0.61	1.29	2.11
MDP0000672573	l-type lectin-domain containing receptor kinase -like	0.0033	12.11	21.35	1.76
MDP0000906869	programmed cell death protein 4-like isoform 1	0.0033	124.90	222.28	1.78
MDP0000668433	tmv resistance protein n-like	0.0034	1.86	3.35	1.80
MDP0000156898	nadph dependent mannose 6-phosphate reductase	0.0035	2.10	8.03	3.83
MDP0000495488	30s ribosomal protein s6 chloroplastic-like	0.0035	5.86	8.96	1.53
MDP0000284863	nadh dehydrogenase	0.0035	14.05	27.58	1.96
MDP0000159661	s-adenosyl-l-methionine-dependent methyltransferases	0.0036	1.23	4.11	3.34

	superfamily protein				
MDP0000785413	expansin-a13-like	0.0036	2.25	5.26	2.34
MDP0000178042	---NA---	0.0037	45.51	72.50	1.59
MDP0000334191	3 -5 exonuclease	0.0037	17.07	29.77	1.74
MDP0000330571	---NA---	0.0038	3.68	8.34	2.26
MDP0000320778	calmodulin-binding transcription activator 2-like	0.0040	0.70	1.73	2.48
MDP0000273754	mitochondrial zinc maintenance protein mitochondrial-like	0.0043	6.98	11.04	1.58
MDP0000910237	bax inhibitor 1-like	0.0043	2.54	6.50	2.56
MDP0000261385	---NA---	0.0044	2.41	4.02	1.67
MDP0000311486	outer envelope pore protein chloroplastic-like	0.0046	4.97	10.11	2.03
MDP0000151022	clathrin light chain protein	0.0047	14.50	23.88	1.65
MDP0000524893	latex-abundant protein	0.0048	13.08	21.27	1.63
MDP0000133079	protein-methionine-s-oxide reductase	0.0048	5.60	11.63	2.08
MDP0000153857	receptor-like protein	0.0049	0.52	1.12	2.16
MDP0000215326	---NA---	0.0050	8.77	13.71	1.56
MDP0000892457	peptidyl-prolyl cis-trans isomerase fkbp20- chloroplastic-like	0.0051	1.83	5.32	2.91
MDP0000191778	serine threonine-protein kinase afc2	0.0052	15.80	29.80	1.89
MDP0000464513	actin-depolymerizing factor family protein	0.0052	5.43	10.91	2.01
MDP0000639264	3 -n-debenzoyl-2 -deoxytaxol n-benzoyltransferase-like	0.0053	1.48	2.64	1.79
MDP0000381157	uncharacterized protein	0.0054	17.40	29.58	1.70
MDP0000145258	subfamily c member 22	0.0057	3.97	7.68	1.93
MDP0000143717	dihydroneopterin aldolase	0.0060	1.15	1.91	1.65
MDP0000690031	sterile alpha motif domain-containing protein	0.0061	4.67	8.18	1.75
MDP0000376244	pyruvate kinase	0.0062	80.59	125.23	1.55
MDP0000128730	nucleoid-associated protein syc1054_d-like	0.0063	16.30	26.63	1.63
MDP0000354002	---NA---	0.0063	2.73	6.01	2.20
MDP0000069846	---NA---	0.0066	11.79	20.41	1.73
MDP0000165052	pre-mrna-splicing factor 38a-like	0.0066	4.26	7.47	1.76
MDP0000928940	hypothetical protein PRUPE_ppa022267mg	0.0069	1.15	2.09	1.82
MDP0000214617	24-sterol c-methyltransferase	0.0074	1.79	3.77	2.11
MDP0000134492	uncharacterized methyltransferase chloroplastic-like	0.0074	2.64	5.12	1.94
MDP0000346600	---NA---	0.0074	4.67	14.18	3.04
MDP0000148258	leucine-rich repeat receptor-like serine threonine-protein kinase at1g17230-like	0.0076	3.90	6.95	1.78
MDP0000847523	acyl-CoA thioesterase	0.0076	5.30	8.86	1.67
MDP0000890649	Major facilitator superfamily protein	0.0076	1.78	2.95	1.66
MDP0000215770	auxin response factor 18-like	0.0078	8.17	12.30	1.50
MDP0000807498	Cytochrome b-c1 complex, subunit 8 protein	0.0079	32.39	49.30	1.52
MDP0000587523	uncharacterized protein	0.0081	2.99	6.39	2.14
MDP0000577338	hypothetical protein PRUPE_ppa010994mg	0.0083	3.05	6.90	2.27
MDP0000599934	heat shock protein binding	0.0083	6.82	13.22	1.94
MDP0000259640	alpha-expansin 13	0.0085	2.67	4.92	1.85
MDP0000317681	ribonuclease p mrp	0.0088	1.05	2.81	2.69
MDP0000231958	60s ribosomal protein l18	0.0089	41.30	62.17	1.51
MDP0000312032	3-hydroxy-3-methylglutaryl coenzyme a reductase	0.0091	21.49	32.33	1.50
MDP0000434532	abscisic acid receptor pyl8-like	0.0091	9.28	19.16	2.06
MDP0000277775	at1g15070 f9l1_1	0.0093	1.43	2.34	1.64
MDP0000262601	pentatricopeptide repeat-containing protein at3g46870-like	0.0093	7.84	11.86	1.51
MDP0000714217	hypothetical protein PRUPE_ppa004877mg	0.0094	2.78	4.25	1.53
MDP0000227470	protein bud31 homolog 2-like	0.0095	2.05	3.64	1.77
MDP0000142029	uncharacterized loc101207989	0.0098	1.59	3.51	2.20
MDP0000175555	b-cell receptor-associated 31-like	0.0100	53.33	82.95	1.56
MDP0000149088	ubiquinone biosynthesis protein coq4	0.0101	3.59	6.55	1.82
MDP0000869501	glutamine dumper	0.0103	1.01	2.75	2.72
MDP0000275302	caffeic acid 3-o-methyltransferase-like	0.0105	44.81	70.93	1.58
MDP0000239632	swi snf-related matrix-associated actin-dependent regulator of chromatin subfamily a member 3-like 3-like	0.0106	0.37	1.09	2.97
MDP0000369845	---NA---	0.0106	9.50	19.29	2.03
MDP0000251022	ac010924_18 ests gb	0.0106	2.19	3.80	1.74
MDP0000269136	vacuolar protein sorting-associated protein 20 homolog 2-like	0.0108	13.01	30.27	2.33
MDP0000807856	protein sensitive to proton rhizotoxicity 1-like	0.0110	9.28	14.53	1.57
MDP0000269476	zinc finger ccch domain-containing protein 24-like	0.0111	0.91	1.97	2.17
MDP0000294250	uncharacterized hydrolase -like	0.0111	1.70	3.16	1.85
MDP0000759534	leucine-rich repeat family protein	0.0111	3.12	5.16	1.65
MDP0000123671	mitochondrial substrate carrier family protein ucpb-like	0.0114	1.94	3.58	1.84
MDP0000339823	---NA---	0.0116	0.46	2.43	5.31
MDP0000143891	probable pre-mrna-splicing factor atp-dependent rna helicase-like isoform x1	0.0117	1.64	3.55	2.16
MDP0000222186	tmv resistance protein n-like	0.0119	0.42	1.54	3.64
MDP0000218521	pentatricopeptide repeat-containing protein at1g74750-like	0.0125	1.99	4.92	2.48

MDP0000339087	---NA---	0.0126	0.70	3.21	4.56
MDP0000300921	cc-nbs-lrr class disease resistance isoform 1	0.0127	1.02	2.00	1.97
MDP0000353802	---NA---	0.0127	2.25	6.08	2.70
MDP0000730224	huntingtin-interacting protein k-like	0.0132	8.92	13.46	1.51
MDP0000539552	polyphenol oxidase ii	0.0135	0.42	1.34	3.19
MDP0000201297	histone-lysine n-methyltransferase suvr2-like isoform x1	0.0135	0.45	1.10	2.42
MDP0000208736	---NA---	0.0137	0.29	1.17	4.09
MDP0000162976	Pectinacetylesterase	0.0140	57.35	86.41	1.51
MDP0000662870	PREDICTED: uncharacterized protein LOC101315376	0.0142	0.45	2.12	4.72
MDP0000263328	ribosome biogenesis protein tsr3 homolog	0.0144	24.20	36.42	1.50
MDP0000508761	flavonol synthase flavanone 3-hydroxylase-like	0.0145	0.57	1.27	2.23
MDP0000141372	crooked neck-like protein 1-like	0.0148	3.82	6.54	1.71
MDP0000146449	cbl-interacting serine threonine-protein kinase 11-like	0.0149	2.03	5.26	2.59
MDP0000769681	histone-lysine n-methyltransferase suvr5-like isoform x1	0.0149	0.15	1.16	7.63
MDP0000263938	arginine serine-rich-splicing factor rsp31-like	0.0151	2.19	5.40	2.46
MDP0000158797	bisphosphoglycerate-independent phosphoglycerate mutase	0.0153	1.67	4.25	2.54
MDP0000847266	e3 ubiquitin-protein ligase atl23-like	0.0155	0.58	1.71	2.95
MDP0000490903	chloroplast biogenesis 19	0.0158	1.26	2.46	1.96
MDP0000843362	histone acetyltransferase hac1-like	0.0158	0.54	2.60	4.86
MDP0000364366	superoxide dismutase	0.0159	19.44	30.52	1.57
MDP0000123286	1-interacting protein	0.0160	1.35	3.36	2.49
MDP0000134766	ubiquinol-cytochrome c reductase complex ubiquinone-binding protein qp-c	0.0164	33.28	50.20	1.51
MDP0000245688	ring-box protein 1a-like	0.0166	4.87	9.56	1.96
MDP0000504159	40s ribosomal protein s19	0.0170	0.45	3.22	7.15
MDP0000685160	inorganic phosphate transporter 2- chloroplastic-like	0.0174	1.32	2.16	1.64
MDP0000193028	phosphatidylinositol- -trisphosphate 3-phosphatase and dual-specificity protein phosphatase	0.0181	1.39	3.40	2.45
MDP0000230147	---NA---	0.0182	8.10	14.05	1.73
MDP0000221593	transcription initiation factor tfiid subunit isoform 1	0.0186	5.21	8.66	1.66
MDP0000449726	60s ribosomal protein l38-like	0.0186	0.92	2.19	2.39
MDP0000346089	---NA---	0.0187	2.33	5.32	2.28
MDP0000827023	uncharacterized protein	0.0199	1.03	2.28	2.22
MDP0000424978	chitinase-like protein 2	0.0201	23.34	36.66	1.57
MDP0000204933	histidine decarboxylase	0.0205	2.50	3.79	1.51
MDP0000221773	protein	0.0210	3.90	11.15	2.86
MDP0000315511	keratin-associated protein 10-6 isoform 1	0.0215	71.76	111.42	1.55
MDP0000392043	---NA---	0.0221	1.10	4.15	3.78
MDP0000142675	---NA---	0.0222	4.70	9.11	1.94
MDP0000543544	ser thr protein kinase	0.0222	4.92	8.52	1.73
MDP0000528745	calcium-transporting atpase plasma membrane-type-like	0.0222	0.60	1.66	2.76
MDP0000278260	uncharacterized loc101214389	0.0223	92.77	153.30	1.65
MDP0000233893	protein n-lysine methyltransferase mettl21a-like	0.0224	1.82	2.89	1.59
MDP0000156903	uncharacterized protein	0.0227	0.77	1.61	2.10
MDP0000211497	nac domain-containing protein 89-like	0.0227	2.58	6.78	2.63
MDP0000356642	uncharacterized protein	0.0228	1.38	4.67	3.39
MDP0000089753	60s ribosomal protein l38	0.0229	0.54	2.48	4.64
MDP0000189737	rna recognition motif-containing protein	0.0232	7.00	11.74	1.68
MDP0000134078	formin-like protein 14-like	0.0233	0.81	1.52	1.88
MDP0000753464	heme-binding-like protein chloroplastic-like	0.0236	10.89	18.47	1.70
MDP0000288574	general transcription factor iih subunit 4-like	0.0242	1.76	2.71	1.54
MDP0000319114	uncharacterized loc101206005	0.0242	8.43	13.00	1.54
MDP0000851476	dna binding	0.0245	0.87	1.80	2.07
MDP0000352879	hypothetical protein EUTSA_v10015648mg	0.0254	1.72	5.91	3.43
MDP0000235803	phospholipase c 3-like	0.0260	0.17	1.13	6.56
MDP0000363637	---NA---	0.0263	1.13	4.03	3.57
MDP0000228681	---NA---	0.0264	0.38	1.04	2.77
MDP0000244352	peptidyl-trna hydrolase mitochondrial-like	0.0264	1.81	3.04	1.68
MDP0000120243	dna polymerase delta subunit	0.0267	0.44	1.77	4.05
MDP0000286180	protein mago nashi homolog isoform 2	0.0267	12.84	19.48	1.52
MDP0000171561	disease resistance protein at3g14460-like	0.0267	2.53	3.81	1.51
MDP0000931239	polyubiquitin	0.0271	5.79	11.64	2.01
MDP0000515577	lsd1-like protein	0.0277	4.76	10.16	2.14
MDP0000169338	uncharacterized loc101207229	0.0280	1.78	3.53	1.99
MDP0000187279	---NA---	0.0285	2.30	5.81	2.52
MDP0000238803	iron-sulfur cluster co-chaperone protein mitochondrial-like	0.0286	2.31	3.91	1.69
MDP0000574105	centromere kinetochore protein zw10 homolog	0.0287	8.60	12.87	1.50
MDP0000149059	PREDICTED: uncharacterized protein LOC101294405	0.0310	3.87	6.80	1.76
MDP0000140187	uncharacterized protein	0.0311	8.93	13.87	1.55
MDP0000265485	cc-nbs-lrr class disease resistance isoform 1	0.0311	1.04	1.85	1.78

MDP0000895282	desacetoxyvindoline 4-	0.0312	1.65	2.57	1.56
MDP0000185841	hypothetical protein PRUPE_ppa019086mg	0.0313	3.92	6.30	1.61
MDP0000375729	hypothetical protein PRUPE_ppa012561mg	0.0314	2.84	5.07	1.78
MDP0000930948	death domain-associated protein 6-like isoform x2	0.0316	19.63	31.05	1.58
MDP0000305834	ruvb-like 2-like	0.0316	1.94	3.03	1.56
MDP0000229317	40s ribosomal protein s30	0.0319	20.54	32.93	1.60
MDP0000361781	---NA---	0.0319	18.28	30.15	1.65
MDP0000119634	dihydrofolate reductase-like	0.0319	4.04	6.07	1.50
MDP0000305441	nudix hydrolase 1-like	0.0322	0.82	1.62	1.97
MDP0000186276	pentatricopeptide repeat-containing protein mitochondrial-like	0.0329	1.29	2.41	1.86
MDP0000460658	histone h3	0.0338	0.14	1.15	7.92
MDP0000146990	ribosomal protein s14	0.0341	5.57	10.75	1.93
MDP0000825981	short life family protein	0.0343	1.81	3.37	1.86
MDP0000208128	2fe-2s ferredoxin	0.0343	2.55	6.35	2.49
MDP0000314632	inorganic carbon transport family protein	0.0344	1.20	2.51	2.10
MDP0000699607	catalase	0.0346	53.52	80.67	1.51
MDP0000123003	hypothetical protein PRUPE_ppa012833mg	0.0380	8.76	20.41	2.33
MDP0000518704	structural constituent of	0.0403	0.91	1.92	2.10
MDP0000171438	auxin-repressed protein	0.0404	8.22	13.47	1.64
MDP0000229720	---NA---	0.0414	2.49	5.03	2.02
MDP0000335839	---NA---	0.0414	15.10	26.12	1.73
MDP0000924771	amme syndrome candidate gene 1 protein homolog	0.0415	5.07	7.67	1.51
MDP0000253936	sodium hydrogen exchanger 2-like	0.0415	2.09	3.51	1.68
MDP0000360549	---NA---	0.0424	19.90	34.12	1.71
MDP0000350340	---NA---	0.0441	0.56	4.39	7.89
MDP0000244450	cdt1-like protein chloroplastic-like	0.0441	0.99	1.67	1.69
MDP0000309881	ring-h2 finger protein atl48-like	0.0443	5.24	8.33	1.59
MDP0000120221	dna ion isoform partial	0.0446	0.46	1.58	3.42
MDP0000266354	---NA---	0.0453	0.38	1.08	2.88
MDP0000808492	cyclin-dependent kinase inhibitor 4	0.0462	0.30	1.24	4.11
MDP0000240941	bag family molecular chaperone regulator 4-like	0.0466	0.73	1.97	2.71
MDP0000190112	serine C-palmitoyltransferase	0.0466	1.36	2.27	1.66
MDP0000732626	wound-induced basic	0.0466	2.69	4.57	1.70
MDP0000303280	biogenesis of lysosome-related organelles complex 1 subunit 2-like	0.0468	13.40	20.44	1.53
MDP0000348754	---NA---	0.0485	1.96	4.68	2.39
MDP0000722904	cyclin-dependent kinase b2-2-like	0.0488	4.17	6.84	1.64
MDP0000908467	---NA---	0.0488	2.44	7.60	3.11
MDP0000293806	acyl CoA oxidase	0.0488	0.75	1.34	1.78
MDP0000212263	uncharacterized membrane protein at3g27390-like	0.0489	4.45	9.79	2.20
MDP0000257595	uncharacterized protein	0.0497	2.10	6.08	2.89
**(B) induced in healthy apples**					
MDP0000209276	ubiquitin-like domain-containing ctd phosphatase-like	<0.0001	3.38	1.54	2.19
MDP0000542815	trna modification gtpase	<0.0001	72.36	46.50	1.56
MDP0000526213	---NA---	<0.0001	1.25	0.10	12.72
MDP0000203927	glutathione peroxidase	<0.0001	49.07	22.51	2.18
MDP0000163886	aconitate hydratase 1	0.0001	34.74	23.14	1.50
MDP0000180017	trna pseudouridine synthase-like	0.0001	1.18	0.32	3.71
MDP0000331084	---NA---	0.0001	13.76	3.33	4.13
MDP0000573529	probable protein phosphatase 2c 60-like	0.0001	577.94	377.14	1.53
MDP0000120718	atp-citrate synthase alpha chain protein 2-like	0.0001	82.25	53.31	1.54
MDP0000186461	alcohol partial	0.0002	5.52	2.33	2.37
MDP0000786706	phosphoenolpyruvate carboxykinase	0.0002	142.87	92.15	1.55
MDP0000240253	glutathione s-transferase omega-like protein	0.0002	1.10	0.39	2.79
MDP0000313282	rna-binding protein nova-1-like	0.0003	113.30	75.78	1.50
MDP0000230483	anaphase-promoting complex subunit 7-like	0.0003	6.71	4.42	1.52
MDP0000619907	dicer-like protein 4	0.0003	35.76	23.53	1.52
MDP0000193534	prenylated rab acceptor family protein	0.0003	13.05	6.71	1.94
MDP0000143103	hypothetical protein PRUPE_ppa003153mg	0.0004	1.11	0.49	2.27
MDP0000194939	chromatin remodeling complex subunit	0.0005	1.59	0.38	4.14
MDP0000314614	lysine--trna ligase-like	0.0005	11.92	6.78	1.76
MDP0000187766	---NA---	0.0006	3.24	1.79	1.81
MDP0000677354	alcohol partial	0.0006	7.30	3.32	2.20
MDP0000172127	ankyrin repeat	0.0006	3.62	1.93	1.88
MDP0000188173	uncharacterized protein	0.0007	1.94	1.15	1.69
MDP0000379515	heat shock protein	0.0007	58.83	33.37	1.76
MDP0000560841	tic22-like family protein	0.0008	2.40	1.14	2.12
MDP0000386580	transcription factor bhlh3-like	0.0010	5.21	2.44	2.13
MDP0000126986	hypothetical protein PRUPE_ppa008026mg	0.0011	49.32	31.79	1.55
MDP0000152903	wd40 domain-containing protein	0.0011	6.70	4.35	1.54

MDP0000350128	---NA---	0.0012	5.13	0.98	5.25
MDP0000790904	hypothetical protein PRUPE_ppa010375mg	0.0013	18.74	9.95	1.88
MDP0000290381	rna-dependent rna polymerase 1-like	0.0014	9.03	4.98	1.81
MDP0000207408	nac domain-containing protein 7-like	0.0016	11.88	4.99	2.38
MDP0000659695	guanine nucleotide-binding protein alpha-2 subunit	0.0017	16.66	10.48	1.59
MDP0000951215	peptide transporter ptr2-like	0.0017	7.01	3.18	2.21
MDP0000448650	copper transporter	0.0020	3.44	1.94	1.77
MDP0000261863	adp-glucose pyrophosphorylase family protein	0.0021	3.20	1.32	2.43
MDP0000225170	protein elc-like	0.0021	2.38	0.96	2.48
MDP0000190479	n-alpha-acetyltransferase 50-like	0.0021	39.73	18.26	2.18
MDP0000252087	uncharacterized pkhd-type hydroxylase at1g22950-like	0.0021	3.79	1.40	2.70
MDP0000127824	spindly family protein	0.0022	6.18	2.68	2.31
MDP0000149770	auxin-induced protein 5ng4	0.0022	1.49	0.68	2.18
MDP0000675290	serine threonine protein phosphatase 2a 59 kda regulatory subunit b zeta isoform-like	0.0023	1.42	0.75	1.90
MDP0000266224	serine-threonine kinase receptor-associated	0.0024	4.59	2.52	1.82
MDP0000775445	DNAJ heat shock family protein	0.0025	23.25	11.16	2.08
MDP0000780347	myosin-11-like isoform x1	0.0025	1.18	0.62	1.92
MDP0000241293	pentatricopeptide repeat-containing protein	0.0025	5.15	2.90	1.78
MDP0000195390	dna primase small subunit-like	0.0025	2.21	0.99	2.23
MDP0000124390	ubiquitin-associated ts-n domain-containing protein	0.0025	13.47	8.70	1.55
MDP0000484420	elongation factor ts family protein	0.0025	4.83	3.02	1.60
MDP0000874407	eukaryotic translation initiation factor 3 subunit m-like	0.0026	2.76	1.17	2.36
MDP0000404090	heat shock 70 kda protein 16-like	0.0026	3.17	1.81	1.75
MDP0000282251	protein breast cancer susceptibility 1 homolog	0.0027	3.11	1.83	1.69
MDP0000185801	deah rna helicase homolog prp2	0.0030	9.66	6.41	1.51
MDP0000547255	bzip transcription factor family protein	0.0031	12.87	8.38	1.54
MDP0000492946	peptidyl-prolyl cis-trans isomerase-like 4-like	0.0031	6.67	4.10	1.63
MDP0000746317	coenzyme Q biosynthesis Coq4 family protein / ubiquinone biosynthesis Coq4 family protein	0.0032	2.04	0.72	2.85
MDP0000218746	pentatricopeptide repeat-containing	0.0033	3.87	1.53	2.53
MDP0000299891	transcription initiation factor tfiid subunit	0.0036	7.94	5.26	1.51
MDP0000677352	probable boron transporter 2-like	0.0037	4.99	1.28	3.90
MDP0000501250	heavy metal atpase	0.0039	79.06	50.78	1.56
MDP0000139791	rna-binding protein 39-like	0.0039	1.18	0.56	2.09
MDP0000314627	cellulose synthase a catalytic subunit 3	0.0041	13.03	8.05	1.62
MDP0000307543	pentatricopeptide repeat-containing protein at3g29230-like	0.0041	4.39	2.49	1.76
MDP0000207712	ribonuclease p protein subunit p25-like	0.0042	7.35	2.01	3.66
MDP0000378491	bifunctional purine biosynthesis	0.0043	5.56	3.17	1.75
MDP0000177522	dnaj heat shock n-terminal domain-containing family protein	0.0044	1.16	0.58	2.01
MDP0000579633	calmodulin-binding protein	0.0045	7.77	4.78	1.63
MDP0000245910	proliferation-associated protein 2g4-like	0.0047	11.58	7.19	1.61
MDP0000881293	uncharacterized loc101205543	0.0049	4.83	2.99	1.62
MDP0000631825	pyruvate kinase isozyme chloroplastic-like	0.0053	13.34	8.44	1.58
MDP0000318625	pho1-like protein	0.0053	20.19	12.86	1.57
MDP0000550900	dead-box atp-dependent rna helicase 39-like	0.0056	9.35	5.71	1.64
MDP0000212560	beta-glucosidase 11-like	0.0057	4.19	2.54	1.65
MDP0000309763	potassium transporter 4-like	0.0057	1.43	0.58	2.47
MDP0000353971	---NA---	0.0060	96.70	61.07	1.58
MDP0000177552	probable f-actin-capping protein subunit beta-like	0.0061	9.04	5.91	1.53
MDP0000315612	tmv resistance protein n-like	0.0061	4.69	2.83	1.65
MDP0000217438	l-galactose-1-phosphate phosphatase	0.0061	17.14	11.24	1.52
MDP0000653993	methionine aminopeptidase 1a-like	0.0062	18.14	10.64	1.70
MDP0000350535	abc transporter g family member 3-like	0.0062	14.66	9.23	1.59
MDP0000723101	nbs-lrr protein	0.0063	1.23	0.62	1.98
MDP0000216731	fom-2 family protein	0.0063	4.07	1.01	4.03
MDP0000260682	inositol pentakisphosphate 2-kinase	0.0064	15.83	9.79	1.62
MDP0000920870	myb family transcription factor apl-like	0.0064	1.80	0.75	2.42
MDP0000347843	acid phosphatase class iiib protein	0.0068	10.19	4.47	2.28
MDP0000122308	aminoacyl-trna hydrolase	0.0068	15.96	9.26	1.72
MDP0000294265	elongation factor 1-alpha-like	0.0069	28.10	12.66	2.22
MDP0000157399	heptahelical transmembrane protein hhp4	0.0069	2.86	1.39	2.05
MDP0000715817	---NA---	0.0070	4.31	2.52	1.71
MDP0000209755	enoyl CoA hydratase	0.0072	4.52	2.71	1.67
MDP0000121191	carboxylesterase 1-like	0.0072	2.28	0.83	2.76
MDP0000360146	---NA---	0.0072	129.63	85.32	1.52
MDP0000229888	probable inactive receptor kinase at5g10020-like	0.0073	1.52	0.54	2.84
MDP0000141789	f-box protein cpr30-like	0.0073	1.89	0.68	2.80
MDP0000501226	vacuolar protein sorting-associated protein 9a-like	0.0075	5.40	3.43	1.58
MDP0000268122	disease resistance protein rga1-like	0.0077	1.68	1.08	1.56

MDP0000309142	f-box lrr-repeat protein 3-like	0.0078	1.83	0.76	2.41
MDP0000564193	at4g02380 t14p8_2	0.0081	10.53	5.88	1.79
MDP0000631061	conserved oligomeric golgi complex subunit 1-like	0.0082	7.39	3.00	2.46
MDP0000286587	pathogenesis related protein partial	0.0084	1.21	0.60	2.01
MDP0000548529	---NA---	0.0085	1.16	0.18	6.52
MDP0000844180	duf21 domain-containing protein at4g14240-like	0.0086	11.83	7.51	1.58
MDP0000249156	disease resistance protein rpm1-like	0.0086	1.45	0.57	2.55
MDP0000284542	ubiquitin-fold modifier-conjugating enzyme 1-like	0.0086	22.70	10.54	2.15
MDP0000551046	protein sensitive to proton rhizotoxicity 1-like	0.0088	11.67	6.19	1.88
MDP0000489997	cobl7 (cobra-like 7)	0.0089	9.32	6.04	1.54
MDP0000162856	keratin-associated protein 10-6 isoform 1	0.0089	48.47	15.83	3.06
MDP0000348668	polycomb group protein curly leaf	0.0091	5.99	3.64	1.65
MDP0000344312	---NA---	0.0092	28.71	16.15	1.78
MDP0000338002	---NA---	0.0093	7.31	2.49	2.93
MDP0000778029	syntaxin-like protein	0.0095	3.78	1.68	2.25
MDP0000141199	nodule-enhanced malate dehydrogenase family protein	0.0096	68.62	45.82	1.50
MDP0000228611	transcriptional activator demeter	0.0099	1.48	0.62	2.40
MDP0000297959	elongation factor 1-alpha-like	0.0102	24.81	12.20	2.03
MDP0000576724	heat repeat-containing protein 5b-like	0.0106	3.84	2.20	1.75
MDP0000184970	---NA---	0.0107	1.46	0.10	13.97
MDP0000322873	---NA---	0.0108	8.38	3.58	2.34
MDP0000316587	copper-exporting p-type atpase a	0.0108	1.91	0.93	2.06
MDP0000936179	valine--trna ligase-like	0.0113	2.84	0.10	28.22
MDP0000196640	ctp synthase-like	0.0113	4.82	2.76	1.75
MDP0000160582	pantothenate kinase-related family protein	0.0114	9.37	5.34	1.76
MDP0000202726	ribulose bisphosphate carboxylase oxygenase activase chloroplastic-like	0.0117	1.97	0.53	3.71
MDP0000224582	eukaryotic translation initiation factor 2a-like	0.0117	12.22	6.71	1.82
MDP0000581903	glyceraldehyde 3-phosphate dehydrogenase	0.0121	142.56	93.35	1.53
MDP0000271929	kinase family protein	0.0123	3.55	1.42	2.49
MDP0000137352	guanylate kinase-like	0.0124	1.39	0.14	10.00
MDP0000555198	mitochondrial import receptor subunit tom7-1-like	0.0126	9.72	4.51	2.16
MDP0000638667	heptahelical protein 4 isoform 2	0.0129	6.44	2.82	2.28
MDP0000267266	proliferation-associated protein 2g4-like	0.0130	12.38	7.55	1.64
MDP0000899645	protein transport protein sec61 gamma subunit	0.0133	3.28	1.31	2.50
MDP0000314952	S-locus lectin protein kinase family protein	0.0138	1.41	0.15	9.35
MDP0000394162	2-oxoglutarate and fe -dependent oxygenase superfamily protein isoform 1	0.0138	5.10	2.18	2.34
MDP0000301957	dna-directed rna polymerase e subunit 1-like	0.0141	3.14	2.10	1.50
MDP0000296000	threonine--trna ligase-like	0.0141	1.63	0.40	4.08
MDP0000176842	tmv resistance protein n-like	0.0143	1.87	0.77	2.42
MDP0000927251	brefeldin a-inhibited guanine nucleotide-exchange protein 2-like	0.0145	12.61	7.56	1.67
MDP0000230065	peptidyl-prolyl cis-trans isomerase-like 4-like	0.0148	6.44	4.15	1.55
MDP0000301638	60s ribosomal protein l18	0.0150	1.78	0.76	2.34
MDP0000316162	probably inactive leucine-rich repeat receptor-like protein kinase at5g48380-like	0.0150	5.64	2.40	2.35
MDP0000912258	hypothetical protein PRUPE_ppb019631mg	0.0150	3.28	1.04	3.16
MDP0000815058	phospholipid-transporting atpase 2-like	0.0151	19.16	12.80	1.50
MDP0000139428	neutral alpha-glucosidase ab-like	0.0151	1.06	0.58	1.82
MDP0000361238	---NA---	0.0152	386.35	237.60	1.63
MDP0000150457	tmv resistance protein n-like	0.0152	2.46	1.03	2.38
MDP0000562741	spindle and kinetochore-associated protein 1 homolog	0.0154	1.33	0.58	2.28
MDP0000898448	---NA---	0.0159	20.57	12.63	1.63
MDP0000168246	atp-citrate synthase alpha chain protein 2-like	0.0165	11.77	7.05	1.67
MDP0000262913	protein dom3z chloroplastic-like	0.0165	8.06	5.33	1.51
MDP0000819246	chitinase 2-like	0.0166	1.70	0.12	14.23
MDP0000127647	uncharacterized loc101214251	0.0166	11.18	7.38	1.52
MDP0000634609	d7-type cyclin	0.0167	3.47	2.00	1.73
MDP0000559078	---NA---	0.0168	10.06	3.59	2.80
MDP0000536398	60s ribosomal protein l6-like	0.0174	2.22	1.03	2.16
MDP0000282442	hypothetical protein PRUPE_ppa009122mg	0.0174	5.92	2.13	2.78
MDP0000151845	endonuclease or glycosyl isoform 1	0.0179	2.54	1.00	2.54
MDP0000352400	---NA---	0.0180	14.58	8.87	1.64
MDP0000306704	f-box kelch-repeat protein at3g06240-like	0.0180	1.23	0.59	2.06
MDP0000848275	dna topoisomerase 1-like	0.0182	18.71	10.80	1.73
MDP0000143894	glutaminyl-peptide cyclotransferase-like	0.0183	6.77	4.44	1.52
MDP0000157996	3-hydroxy-3-methylglutaryl coenzyme a reductase	0.0183	3.72	1.94	1.92
MDP0000322233	peptide methionine sulfoxide reductase b5	0.0183	11.02	5.79	1.90
MDP0000633537	40s ribosomal protein s6-like	0.0187	6.28	3.05	2.06
MDP0000399958	ubiquitin-conjugating enzyme e2-21 kda 3 family protein	0.0190	2.32	1.18	1.97

MDP0000151679	ribonuclease p mrp	0.0190	1.36	0.53	2.56
MDP0000693473	yippee-like family protein	0.0190	3.84	0.87	4.41
MDP0000366240	ubiquitin-like protein 5-like	0.0191	24.66	11.43	2.16
MDP0000204619	cyclin-dependent kinase d-1-like	0.0191	9.59	3.79	2.53
MDP0000276629	glycerol-3-phosphate acyltransferase 3-like	0.0193	19.90	9.23	2.16
MDP0000731864	pentatricopeptide repeat-containing protein at1g74750-like	0.0194	9.41	6.18	1.52
MDP0000250207	tesmin tso1-like cxc domain-containing isoform 2	0.0202	2.41	1.14	2.11
MDP0000207866	uncharacterized loc101207235	0.0204	10.38	6.53	1.59
MDP0000290990	abc transporter e family member 2-like	0.0210	17.61	11.67	1.51
MDP0000141576	vesicle-associated membrane protein 727	0.0212	15.57	9.83	1.58
MDP0000200406	p17 29c-like protein ddb_g0287399-like	0.0212	38.63	19.55	1.98
MDP0000241761	protein unc-50 homolog	0.0215	3.11	1.40	2.22
MDP0000130930	hypothetical protein PRUPE_ppa011746mg	0.0216	1.83	0.87	2.12
MDP0000231836	---NA---	0.0219	2.55	1.41	1.81
MDP0000166076	las1-like protein	0.0227	2.40	0.81	2.97
MDP0000193649	---NA---	0.0231	1.44	0.48	3.00
MDP0000203462	bes1 bzr1 homolog protein 2-like	0.0232	12.37	7.82	1.58
MDP0000943413	lrr and nb-arc domains-containing disease resistance	0.0233	41.45	26.44	1.57
MDP0000459816	disease resistance rpp13-like protein 1-like	0.0234	5.74	3.64	1.58
MDP0000155389	mads box	0.0236	12.03	7.61	1.58
MDP0000218596	20s proteasome beta subunit pbb2	0.0248	4.96	2.81	1.76
MDP0000309417	aldo-keto reductase family 4 member c9-like	0.0264	6.03	3.81	1.58
MDP0000272705	patatin t5	0.0268	5.19	2.31	2.24
MDP0000819586	late embryogenesis abundant domain-containing family protein	0.0269	2.13	1.08	1.97
MDP0000585959	splicing factor 3b subunit 3-like	0.0270	1.03	0.69	1.51
MDP0000168441	probable u3 small nucleolar rna-associated protein 11-like	0.0274	10.04	4.71	2.13
MDP0000692555	adrenodoxin-like mitochondrial-like	0.0275	4.32	1.21	3.57
MDP0000235992	probable s-acyltransferase at4g24630-like	0.0278	5.33	3.05	1.75
MDP0000422184	sphingosine-1-phosphate lyase	0.0279	10.76	6.40	1.68
MDP0000266629	uncharacterized protein	0.0282	1.48	0.99	1.50
MDP0000305960	uncharacterized protein loc100276021	0.0288	11.06	5.88	1.88
MDP0000606291	zf-4cxxc-r1 transcription factor and jumonji domain-containing protein	0.0289	1.84	0.81	2.28
MDP0000295666	O-fucosyltransferase family protein	0.0297	2.39	1.45	1.65
MDP0000200226	O-fucosyltransferase family protein	0.0297	2.39	1.45	1.65
MDP0000798289	copper-exporting p-type atpase a	0.0299	1.53	0.79	1.94
MDP0000176263	actin cytoskeleton-regulatory complex protein pan1-like	0.0306	6.63	3.66	1.81
MDP0000259111	dna-binding protein gt-1	0.0306	17.49	10.99	1.59
MDP0000937193	pollen-specific leucine-rich repeat extensin-like protein 2-like	0.0306	32.71	16.13	2.03
MDP0000208497	regulatory protein	0.0308	6.81	3.56	1.91
MDP0000342781	---NA---	0.0316	9.67	5.21	1.86
MDP0000391052	---NA---	0.0318	2.55	1.66	1.54
MDP0000476444	calmodulin 24-like protein	0.0321	9.32	5.40	1.73
MDP0000356903	act domain-containing protein	0.0325	5.51	0.91	6.03
MDP0000500503	tmv resistance protein n-like	0.0334	1.83	1.11	1.64
MDP0000126801	hypothetical protein PRUPE_ppa012278mg	0.0334	3.00	1.90	1.58
MDP0000138583	lrr receptor-like serine threonine-protein kinase gso1-like	0.0342	1.71	1.14	1.50
MDP0000545900	tmv resistance protein n-like isoform x4	0.0344	2.98	1.74	1.71
MDP0000203780	c2h2 and c2hc zinc fingers superfamily protein	0.0344	1.43	0.62	2.32
MDP0000158136	indeterminate -domain isoform 2	0.0353	1.19	0.63	1.90
MDP0000573939	profilin	0.0362	120.12	66.29	1.81
MDP0000175647	serine threonine-protein phosphatase 2a regulatory subunit b subunit alpha-like	0.0364	9.19	5.94	1.55
MDP0000516523	---NA---	0.0367	18.27	9.81	1.86
MDP0000188922	cysteine-rich receptor-like protein kinase 29-like	0.0371	2.34	0.78	2.99
MDP0000124910	ribosome biogenesis protein bms1-like protein	0.0372	3.50	1.92	1.82
MDP0000589360	importin-5- partial	0.0383	2.28	0.79	2.89
MDP0000044410	elongation factor 1-alpha	0.0387	7.55	4.33	1.74
MDP0000743397	pyruvate kinase cytosolic isozyme-like	0.0390	12.05	6.33	1.90
MDP0000245646	40s ribosomal protein s23-like	0.0403	4.95	3.06	1.62
MDP0000636876	tmv resistance protein	0.0434	1.26	0.60	2.11
MDP0000125112	phospholipid-transporting atpase 9-like	0.0449	1.13	0.53	2.15
MDP0000278354	epsin n-terminal homology domain-containing family protein	0.0452	5.67	3.62	1.57
MDP0000312965	disease resistance protein	0.0460	1.39	0.42	3.29
MDP0000342785	---NA---	0.0465	10.10	5.78	1.75
MDP0000946509	geranylgeranylated protein atgp4	0.0474	1.57	0.12	13.44
MDP0000336518	e3 ubiquitin-protein ligase rha1b-like	0.0475	3.30	1.26	2.61
MDP0000119941	dihydrolipoyl dehydrogenase 2	0.0475	3.46	1.76	1.97
MDP0000608579	eukaryotic translation initiation factor 4e	0.0487	2.58	1.33	1.94

Table S5. Primer sequences used for validation of RNA-Seq results by qRT-PCR.

Gene name	*Malus* name	Malus gene ID	Forward primer	Reverse primer	PCR product (bp)
phospholipase a2	*MdPLA2*	MDP0000249250	CGGAAGAATGTTATCTTGGTAGGT	GGAGCTTGAGTTCGTCTTTATTTC	102
L-ascorbate oxidase	*MdAO*	MDP0000610961	CTTAGAGATGCAGTGTCCAGAAGT	CATCCTCATTTACGGTTTTAGTCTC	96
polyphenol oxidase	*MdPPO*	MDP0000249183	CTAGGAATATACGGCGGATCAA	GTCCAGAGTGATCGGTAACTCACT	120
chorismate mutase/ascorbate peroxidase	*MdCM*	MDP0000196554	CGCGCTCTTAGTCTGTATTCTTT	CGTAAGTTTTCGCCTTCATTTC	122
xyloglucan endotransglucosylase/hydrolase	*MdXET*	MDP0000873667	CCACAAAAACCTTCCACACATAC	GTGACATGGATTCTGACCTAAAGA	113
pyruvate dehydrogenase	*MdPDH*	MDP0000192364	TTAGCTGATTACGAAAGAGGAAGG	GCCATTGTAAAGGTGAACGAAG	130
1-aminocyclopropane-1-carboxylate oxidase	*MdACO*	MDP0000200896	GTGGAAGTGCTGAGTAATGGAAG	AGGTAATCTTGAAAACGGAGGTG	170
cellulose synthase	*MdCesA*	MDP0000289339	AGGTTTGTTTGGAGAGGGTAAA	AGGATGTAGTGTTCTTAGGGCAGA	107
polyphenol oxidase	*MdPPO-2*	MDP0000539552	GGACATCGAGTTTGAGAGCAAC	TGACTTGTTTTTAGGCCAGTCTTC	150
pyruvate kinase	*MdPK*	MDP0000376244	AGTACCATCAGGAGACCCTCAAC	GGAAGGATGAAAGGAGAGAAGAA	114
3-hydroxy-3-methylglutaryl coenzyme a reductase	*MdHMGR*	MDP0000312032	AGTGAAGGGTGCTAACAGAGAGG	GCTGAAATAGCAGACATGAGAGAA	111
succinate dehydrogenase	*MdSDH*	MDP0000251581	CCCTCATTGCTGCTAATTGGAT	GTAGCCTGGAAAAACCAAAAGTG	199
glutathione peroxidase	*MdGPX*	MDP0000203927	CATTTACAAGGGAAAGTTCTGCTG	CTCATCGTAATTTGAGTTGGTCAG	158
L-galactose-1-phosphate phosphatase	*MdGPP*	MDP0000217438	GACTATTGTGTGGGATAATGTGGA	GACGTGAGATTTGTAGAGGATGG	135
glycerol-3-phosphate acyltransferase	*MdGPAT*	MDP0000276629	GCAAGAGATAGTTTGACTGGATGA	CACTGGAATGAAGGATGATAGGA	160
ubiquitin	*MdUBQ*	MDP0000154072	TCCTCACGTCTATCCAGTCACTAC	TTAGTCTGCTGTCCAACTCTGTTC	146
actin	*MdACT*	MDP0000886327	GATGGCTACTCATTTCCTCATCTT	TTCTCCTTGATTTCCCTAACAGTC	146

Table S6. Gene expression values (RPKM) of the different dehydroascorbate reductases (*DHAR*) as revealed by RNA-Seq. Different letters indicate significant differences ($P < 0.05$) between values based on Tukey's test in SAS.

	Malus ID	harvest		healthy		affected		harvest		healthy		affected	
				inner cortex						outer cortex			
DHAR1	MDP0000127419	30.50	a	9.06	b	6.70	b	31.42	a	8.44	b	10.23	b
DHAR2	MDP0000146156	1.41	a	3.76	b	2.14	b	0.96	a	1.74	a	1.21	a
DHAR3	MDP0000156763	0.21	a	0.00	a	0.02	a	0.06	a	0.00	a	0.06	a
DHAR4	MDP0000175246	0.22	a	0.06	a	0.02	a	0.06	a	0.00	a	0.05	a
DHAR5	MDP0000236168	76.57	a	40.76	b	48.14	b	93.66	a	53.07	b	50.85	b
DHAR6	MDP0000240690	6.84	a	0.30	b	1.43	b	9.47	a	1.20	b	0.92	b
DHAR7	MDP0000311865	0.25	a	0.90	a	0.55	a	0.09	a	1.79	a	1.17	a
DHAR8	MDP0000316839	36.14	a	12.78	b	16.80	b	43.04	a	13.96	b	18.15	b
DHAR9	MDP0000530903	20.46	a	12.54	b	12.44	b	21.83	a	15.33	b	14.95	b
DHAR10	MDP0000942136	1.66	a	2.51	b	1.36	b	1.00	a	1.16	a	1.51	a

Table S7. The browning index (BI) of inner and outer cortex of apples used for RNA-Seq. Apples have been stored for four months at controlled storage conditions. Results represent the mean BI of the five inner or outer cortex pieces (Additional file 2: Figure S1) of healthy and affected tissues ± standard deviation (*n* = number of observations). The colour scale used for browning assessment is based on ten classes ranging from yellow (BI = 1) to brown (BI = 10).

	**Browning Index**	
**classification**	**inner cortex**	**outer cortex**
healthy	3.18 ± 0.29 (*n*=20)	3.76 ± 0.23 (*n*=40)
affected	4.78 ± 0.34 (*n*=60)	4.81 ± 0.35 (*n*=40)

Table S8. The browning index (BI) of inner and outer cortex of the validation set. Apples were stored for two, four, or six months under various controlled atmosphere conditions. Based on their BI, tissues were classified as healthy (low BI) and affected (high BI). Results represent the mean BI of the five inner or outer cortex tissues of five apples ± standard deviation. The colour scale used for browning assessment is based on 10 classes ranging from yellow (BI = 1) to brown (BI = 10).

			Browning index	
**Storage time (months)**	**Conditions**	**Cortex classification**	**inner cortex**	**outer cortex**
2	1% O_2, 3.7% CO_2, commercial application of triazole and 1-MCP	healthy	3.60 ± 0.68	3.79 ± 0.57
		affected	5.77 ± 0.54	4.79 ± 0.41
4	1% O_2, 3.7% CO_2, no application of triazole and 1-MCP	healthy	3.89 ± 0.15	4.11 ± 0.15
		affected	4.40 ± 0.22	4.62 ± 0.28
6	3% O_2, 0.7% CO_2, no application of triazole and 1-MCP	healthy	3.59 ± 0.51	3.91 ± 0.58
		affected	5.04 ± 0.40	4.97 ± 0.26
